# Development of HPV16 mouse and dog models for more accurate prediction of human vaccine efficacy

**DOI:** 10.1186/s42826-023-00166-3

**Published:** 2023-06-12

**Authors:** Emmanuelle Totain, Loïc Lindner, Nicolas Martin, Yolande Misseri, Alexandra Iché, Marie-Christine Birling, Tania Sorg, Yann Herault, Alain Bousquet-Melou, Pascale Bouillé, Christine Duthoit, Guillaume Pavlovic, Severine Boullier

**Affiliations:** 1 INTHERES, INRAE, ENVT, Université de Toulouse, Toulouse, France; 2 CNRS, INSERM, CELPHEDIA, PHENOMIN-Institut Clinique de la Souris (ICS), Université de Strasbourg, 1 rue Laurent Fries, 67404 Illkirch Graffenstaden, France; 3 FlashTherapeutics, Centre de Recherche Langlade, 3 Avenue Hubert Curien, 31100 Toulouse, France; 4 CNRS, INSERM, Institut de Génétique et de Biologie Moléculaire et Cellulaire (IGBMC), Université de Strasbourg, 1 rue Laurent Fries, 67404 Illkirch Graffenstaden, France; 5 Genticel, Toulouse, France

**Keywords:** HPV, Preclinical research, Immunology, Vaccine validation, Canine model, Genetically modified mouse tools

## Abstract

**Background:**

Animal models are essential to understand the physiopathology of human diseases but also to evaluate new therapies. However, for several diseases there is no appropriate animal model, which complicates the development of effective therapies. HPV infections, responsible for carcinoma cancers, are among these. So far, the lack of relevant animal models has hampered the development of therapeutic vaccines. In this study, we used a candidate therapeutic vaccine named C216, similar to the ProCervix candidate therapeutic vaccine, to validate new mouse and dog HPV preclinical models. ProCervix has shown promising results with classical subcutaneous murine TC-1 cell tumor isografts but has failed in a phase II study.

**Results:**

We first generated E7/HPV16 syngeneic transgenic mice in which the expression of the E7 antigen could be switched on through the use of Cre–lox recombination. Non-integrative LentiFlash^®^ viral particles were used to locally deliver Cre mRNA, resulting in E7/HPV16 expression and GFP reporter fluorescence. The expression of E7/HPV16 was monitored by in vivo fluorescence using Cellvizio imaging and by local mRNA expression quantification. In the experimental conditions used, we observed no differences in E7 expression between C216 vaccinated and control groups. To mimic the MHC diversity of humans, E7/HPV16 transgenes were locally delivered by injection of lentiviral particles in the muscle of dogs. Vaccination with C216, tested with two different adjuvants, induced a strong immune response in dogs. However, we detected no relationship between the level of cellular response against E7/HPV16 and the elimination of E7-expressing cells, either by fluorescence or by RT-ddPCR analysis.

**Conclusions:**

In this study, we have developed two animal models, with a genetic design that is easily transposable to different antigens, to validate the efficacy of candidate vaccines. Our results indicate that, despite being immunogenic, the C216 candidate vaccine did not induce a sufficiently strong immune response to eliminate infected cells. Our results are in line with the failure of the ProCervix vaccine that was observed at the end of the phase II clinical trial, reinforcing the relevance of appropriate animal models.

**Supplementary Information:**

The online version contains supplementary material available at 10.1186/s42826-023-00166-3.

## Background

Human papillomaviruses (HPV) are the second most important infectious agent worldwide after *Helicobacter pylori* (770,000 cases). Each year, they are responsible for approximately 640,000 cancer-causing infections [1, 2]. Chronic infection with HPV types is causally associated with mostly anogenital cancers, as well as multiple head and neck squamous cell subtypes, in particular oropharyngeal cancer [3]. Cervical carcinoma alone is the fourth most common cancer in women worldwide [4]. Of the 15 oncogenic genital HPV types, HPV16 is the most common, followed by HPV18 and HPV45, which contribute to approximately 50%, 20%, and 10%, of cervical cancer cases, respectively [5].

In the natural immune response against HPV, E6- and E7-specific T-helper cells have a role in the control of HPV and in the elimination of HPV-infected cells [6, 7]. Interestingly, these proteins are expressed from the very early stage of infection and remain present even after oncogenic cell transformation [8]. The E6 and E7 proteins are therefore interesting candidates for the development of a therapeutic vaccine.

C216 is a new trivalent E7/HPV therapeutic candidate vaccine that is very similar to ProCervix (also called GTL001), a vaccine candidate that failed in a phase II clinical trial (ClinicalTrials.gov: NCT01957878). Both candidate vaccines are based on a recombinant detoxified adenylate cyclase toxin (CyaA) initially isolated from *Bordetella pertussis* hemolysin [9]. The C216 compound is composed of a detoxified and modified CyaA named Vaxicalse carrying fused non-oncogenic E7/HPV-16, E7/HPV-18 and E7/HPV-45 antigens, whereas ProCervix contains a combination of detoxified CyaAs carrying non-oncogenic E7/HPV-16 and E7/HPV-18 antigens [10, 11]; EP2875130B1. By binding to the highly conserved CD11b/CD18 integrin receptor with high affinity [12], CyaA targets immune cells that specifically express CD11b/CD18, in particular antigen-presenting cells. After binding, CyaA is translocated into the cell cytoplasm where it initiates a specific CD8 T cell response. By inserting antigens in place of its catalytic domain, it has been shown that CyaA can deliver simultaneously several epitopes without loss of immunogenicity [13]. In addition, this system also leads to antigen presentation by the major histocompatibility complex (MHC) class II pathway [14].

HPV presents very narrow host specificity and is only known to infect primates [15]. The wealth of knowledge relating to the biology of the virus relies almost exclusively on non-human papillomavirus experimentation [16]. As observed with the COVID-19 pandemic [17], the paucity of suitable preclinical animal models is a major obstacle to the development of specific therapeutics. Existing transgenic mouse lines are not sufficient for preclinical research on HPV therapeutic vaccines. In K14-E7/HPV16 mice, the oncogenic E7 protein is expressed constitutively in the skin [18]. These mice are thus tolerant to the antigen, which consequently leads to no immune responses, so it is necessary to first break the immune tolerance before they can be used effectively. The TC-1 murine E7/HPV16-expressing tumor model [19] is the most classically used preclinical tool for therapeutic HPV vaccine research [20]. TC-1 cells are derived from C57BL/6N mice and can therefore only be used in this genetic background: this mouse strain does not represent the complexity of the cell types that can be transformed by HPV. This model can therefore only be used in preliminary studies. In addition, similar cell lines that express E6 or E7 antigens from other HPV serotypes (for example HPV18 or HPV45) are unavailable, restricting the field of use of these cellular models to HPV16 serotype studies.

The main objective of this study was to develop new animal models to evaluate the immune response and the efficacy of candidate vaccines against HPV infections. Two complementary approaches were tested to obtain animals expressing the HPV E7 antigen using two different animal models, mouse and dog. We first generated E7/HPV16 transgenic mice in which the expression of the E7 antigen could be switched on through the use of Cre–lox recombination. Non-integrative LentiFlash^®^ viral particles [21] were used to locally deliver Cre mRNA, resulting in E7/HPV16 expression and green fluorescent protein (GFP) reporter fluorescence. For our second model, we selected Beagle dogs because the canine immune system and immune responses are, in general, very similar to those of humans [22, 23] and the CyaA protein can bind to canine monocytes and induce an IFNγ response in dogs ([24], unpublished data). We delivered E7/HPV16 transgenes to dogs by intramuscular (IM) injection of lentiviral particles. The characterization, in dogs, of the local and systemic safety as well as the immune response induced, in particular that of cytotoxic T lymphocytes associated with a Th1-type immune response, has allowed for better preclinical evaluation, which is necessary for the development of a new vaccine candidate for human therapy. These two models were used to test the efficacy of the C216 compound, a therapeutic candidate vaccine against HPV16, HPV18, and HPV45 infections.

## Results

### Generation and validation of an E7/HPV16-eGFP mouse model

**Fig. 1:**
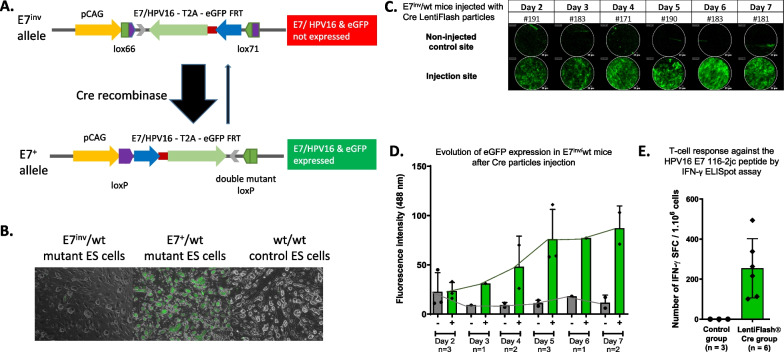
Generation of the E7/HPV16-eGFP conditional mouse model. **A** Schematic drawing of the targeting strategy. Before Cre-mediated recombination, the E7/HPV16-eGFP allele (E7^inv^ allele) is in antisense from the pCAG promoter. After Cre-mediated recombination, the E7/HPV16-eGFP is expressed (E7^+^ allele). *Lox66* and *lox71* were used to reduce Cre-mediated reversion from the E7 allele to the E7^inv^ allele [55]. **B** The expression of the E7/HPV16-eGFP construct was validated by GFP fluorescence before (E7^inv^/wt) and after (E7^+^/wt) Cre recombinase-mediated inversion in mutant ES cells and wild-type ES control cells (wt/wt). **C** IM injection of Cre LentiFlash^®^ was performed in E7^inv^/wt mice at day 0 to locally induce E7/HPV16-eGFP expression. The detection of the local expression of green fluorescence was realized with the MiniZ probe using the Cellvizio^®^ system at 488 nm, from day 2 to 7. One image was extracted from each video to visualize the fluorescence expression at the injected point in comparison with the non-injected (NI) point. **D** The mean fluorescence intensity values (± SD) are shown. The fluorescence intensity was calculated as the mean of the fluorescence intensity of the totality of every point captured during the video for each animal (individual dot). When several mice were tested at the same time, the means of the results for each mouse were calculated. **E** The mean ± SD of the number of IFN-y SFC per 10^6^ cells is shown for both control and Cre LentiFlash^®^ Cre mouse groups. Splenocytes were collected from the NI mice (control group) and at 8 days after Cre particle injection (Cre LentiFlash^®^ group) and were stimulated in triplicate to evaluate the T-cell response against the HPV16 E7 116-2jc peptide by IFN-y ELISpot assay. For each mouse, the number of spot-forming cells (SFC) corresponds to the median of triplicates and was corrected with the negative control. One-tailed Wilcoxon test was used (*p value < 0.05)

Existing animal models that mimic E7 cell expression after HPV infection are inadequate, which hampers testing and validation of new HPV therapies [16]. To overcome the defects of the existing models, we developed a new mouse line that conditionally expresses E7/HPV16 peptide using C57BL/6N embryonic stem (ES) cells. We used the E7/HPV16 sequence described in Esquerre et al. [25] because of the demonstrated immunogenicity of the corresponding peptides [26]. This E7 sequence was inserted in the *Rosa26* neutral locus under the control of the CAG promoter to allow an expression of E7 in a large number of mouse cells (Fig. 1A). An eGFP sequence was fused with a T2A sequence (encoding a self-cleaving peptide [27]) to the E7/HPV16 sequence to allow the detection of expressing cells by fluorescence. Before Cre-mediated recombination, the E7/HPV16-eGFP sequence was in antisense from the CAG promoter (E7^inv^ allele) and was not expressed in modified ES cells (Fig. 1B). After Cre-mediated recombination, the E7/HPV16-eGFP sequence (E7^+^ allele) was strongly expressed as visualized by eGFP fluorescence in ES cells and RT-qPCR data (Fig. 1B). E7^inv^/wt modified ES cells were then injected into blastocysts to generate the corresponding mouse model. Efficient in vivo Cre recombination of the E7^inv^ allele into the E7^+^ allele was verified by crossing E7^inv^/wt mice to the ubiquitous C57BL/6NTac-Gt(ROSA)26Sor^tm2(CAG−flpo,−EYFP)Ics^ Cre deleter (data not shown; [28]). To mimic HPV16 infection, we injected in the right gastrocnemius muscle, non-integrative Cre LentiFlash^®^ particles as a means to locally deliver the Cre mRNA. This approach allowed transient expression of the Cre recombinase, without any lentivector integration in the genome [21]. The eGFP expression was then followed, for up to 35 days, by in vivo fluorescence analysis using Cellvizio imaging (Fig. 1C, D) and by RT-ddPCR. In non-injected muscle, only the background expression was observed, whereas in muscle injected with Cre LentiFlash^®^ particles, eGFP was significantly expressed starting at day 4 (Fig. 1C, D). Using an ELISpot assay, we demonstrated that the expression of E7/HPV16 protein in Cre LentiFlash^®^ injected E7^inv/^wt mice induced a specific T-cell response against E7 from HPV16, 8 days after Cre induction. This immune response was not observed in non-injected E7^inv/^wt mice (Fig. 1E). We were thus able to generate a genetically modified line in which E7 expression and immunogenicity against E7 could be locally induced by Cre-mediated recombination.

### Evaluation of the candidate vaccine prophylactic efficacy using E7^inv^/wt mice

**Fig. 2:**
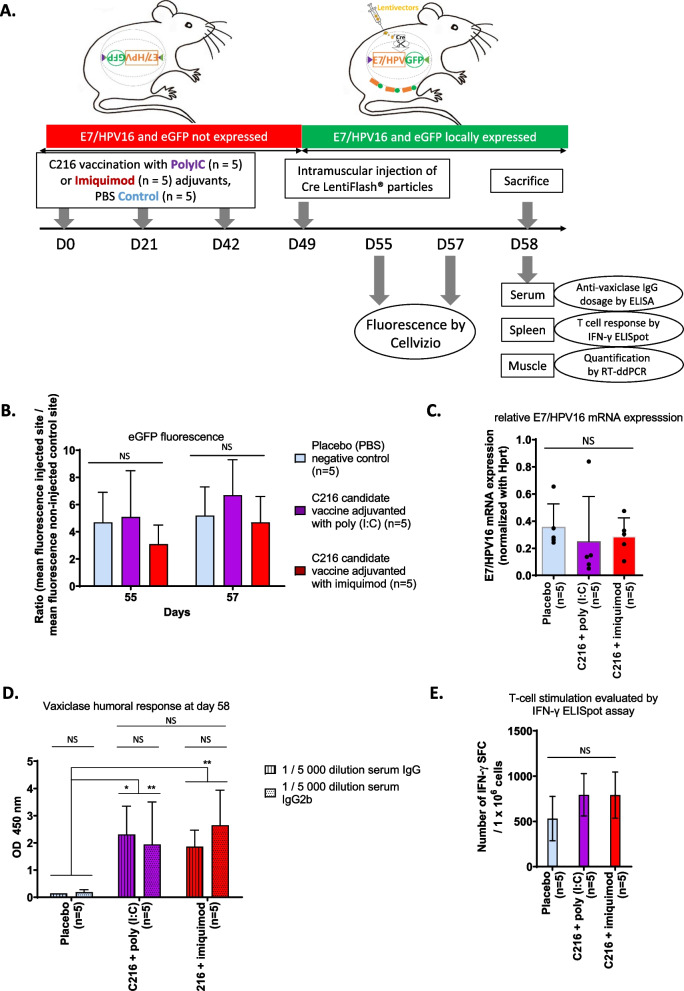
Evaluation of the efficacy of compound C216 as a prophylactic vaccine in E7^inv^/wt mice. **A** Experimental design. **B** The fluorescence was acquired using the Cellvizio imaging system with the MiniZ probe at 488 nm. Background fluorescence was corrected by comparing with that at the non-injected control site, for every mouse at days 55 and 57. For each injection point, fluorescence intensity was evaluated with a 30–45 s laps video. Each image of the video was used to calculate the mean fluorescence intensity of each injection point. The corrected fluorescence was calculated according to a ratio between the mean of the fluorescence intensity at the lentiviral injection point and the mean of the fluorescence intensity at the NI point. Finally, the geometric mean of the ratio ± SD was calculated for each group. **C** E7/HPV16 mRNA was quantified by RT-ddPCR. Expression was normalized with *Hprt* housekeeping gene as described in [53]. **D** Total serum IgG and lgG2 were measured by ELISA, the optical density (OD) was obtained at 450 nm and the means of the ODs ± SD were calculated. **E** T-cell stimulation was evaluated by IFN-γ ELISpot assay. Splenocytes were collected from mice, 9 days after lentiviral injection and stimulated in triplicate with the HPV16 E7 116-2jc peptide. For each mouse, the number of spot-forming cells (SFC) corresponds to the median of the triplicate, corrected with the negative control (DMSO). The mean ± SD of the number of IFN-γ SFC per 10^6^ cells was calculated for the 3 groups. Differences between groups were determined using the Mann–Whitney statistical test. SD: Standard deviation, NS: Non-significant, *p value < 0.05; ** p value < 0.01

To evaluate the efficacy of the C216 candidate vaccine, E7^inv^/wt mice were vaccinated with three consecutive intradermal injections 21 days apart. Two different conditions were compared to a placebo negative control (PBS): C216 candidate vaccine adjuvanted with imiquimod or adjuvanted with poly (I:C). We found that the peak of the candidate vaccine-induced specific immune response was observed 7 days after the third injection (data not shown). At this time point (day 49), an IM injection of Cre LentiFlash^®^ particles was performed to locally induce the expression of E7/HPV16 protein (Fig. 2A). Fluorescence expression of eGFP was monitored 6 (day 55) and 8 (day 57) days after injection of Cre LentiFlash^®^ particles by Cellvizio measurement (Fig. 2B). The mean ratio of fluorescence was not statistically different between the three groups and was stable between the two sets of measures. These data were confirmed by RT-ddPCR quantification of E7/HPV16 mRNA at the Cre LentiFlash^®^ particle injection site. There was no significant difference between the three groups (Fig. 2C). In our experimental design, these results highlight that C216 compound adjuvanted with imiquimod or poly (I:C) was not able to reduce E7 expression.

To understand the inefficiency of the C216 compound as a prophylactic vaccine, we then explored whether this C216 candidate vaccine could induce an efficient immune response in our mouse model. We first determined the profile of the humoral immune response against Vaxiclase, the vaccine vector (Fig. 2D). We measured a strong humoral immune response against Vaxiclase in both vaccinated groups and, as expected, no response was detected in the control group. Furthermore, no significant difference could be observed depending on the adjuvant. Interestingly, we observed in both vaccinated groups the synthesis of Vaxiclase-specific IgG2b antibodies, humoral markers of a Th-1 response (Fig. 2D).

We therefore checked whether the C216 compound induced a cellular response against E7/HPV16 protein. IFNγ Elispots specific for a pool of E7/HPV16 peptides were performed as described in [11] (Fig. 2E). As observed in section 3.1, an E7/HPV16 T-cell response was measured in the control group locally expressing the E7/HPV16 protein after injection of Cre LentiFlash^®^ particles. However, in both experimental groups vaccinated with C216 candidate vaccine adjuvanted with imiquimod or adjuvanted with Poly (I:C), these three consecutive prophylactic vaccinations did not improve significantly the E7/HPV16 T-cell response compared to the control group.

### Evaluation of the candidate vaccine prophylactic efficacy in dogs

To confirm these results, we then evaluated the candidate vaccine in beagle dogs.

**Fig. 3:**
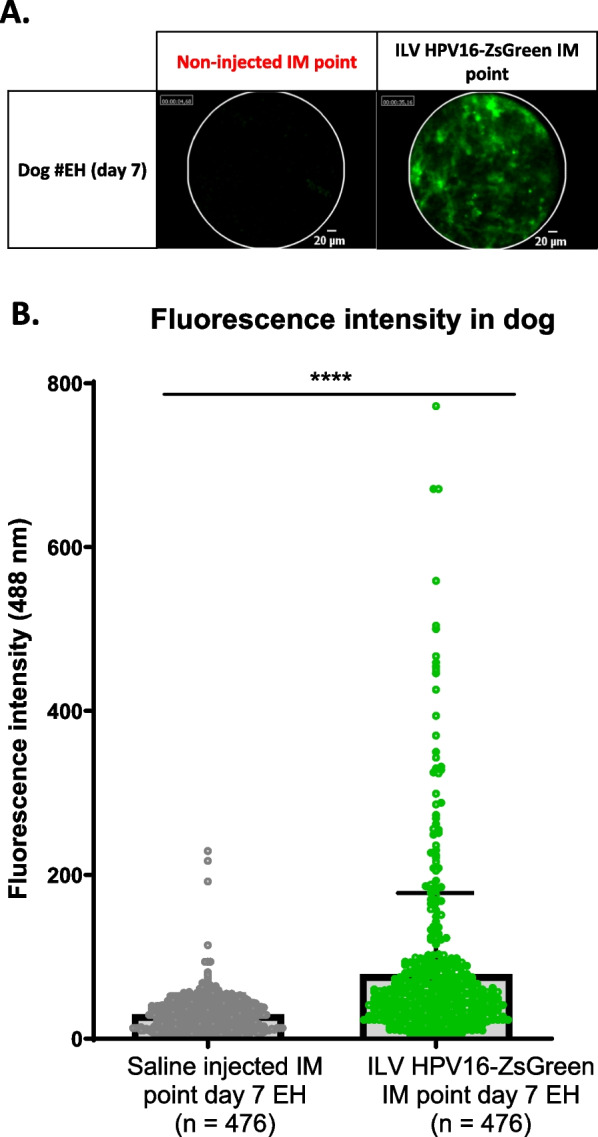
Expression of ZsGreen fluorescence using the ILV HPV16-ZsGreen lentivector in dog muscle. Dogs were injected in muscle at day 0. The detection of the local expression of green fluorescence was realized with the MiniZ probe using the Cellvizio^®^ imaging system at 488 nm at day 7. **A** A representative image was extracted from each video to visualize the fluorescence expression at the injected point in comparison with the non-injected point. **B** The evaluation of the fluorescence intensity at the vector-injected point and the saline-injected point was performed at day 7 post-injection. For each point, the fluorescence intensity was evaluated with a 30–45 s laps video. The fluorescence intensity of each image of the video (n = 476) was recorded and used to calculate the mean fluorescence intensity of each injection point. The fluorescence intensity was expressed as the mean ± SEM of the fluorescence intensity of each site. SEM = standard error of the mean. **p value < 1%

Prior to testing in dogs, in an effort to minimize the number of dogs used in this experiment, the expression of E7/HPV16-ZsGreen ILV was first set up and confirmed in C57BL/6N mice (Additional file 1: Fig. S1) and then verified in a single dog (Fig. 3A, B) by quantifying ZsGreen fluorescence using the Cellvizio imaging system. To mimic HPV infection, an integrative lentivector (called E7/HPV16-ZsGreen ILV), which encodes the E7/HPV16 protein and the fluorescent reporter ZsGreen, was injected intramuscularly (IM). As E7/HPV16 and ZsGreen were linked by a T2A self-cleaving peptide [29], detection of ZsGreen protein indicated the presence of the E7/HPV16 protein or a fusion between E7/HPV16 and ZsGreen. Seven days after E7/HPV16-ZsGreen ILV, an easily detectable fluorescence was observed at the vector injection site (Fig. 3A) in the dog muscle. The comparison of the fluorescence intensity in saline and vector-injected sites showed a significant fluorescence expression at the vector injection site (Fig. 3B), indicating that the E7/HPV16-ZsGreen ILV vector could be used in dogs.

**Fig. 4:**
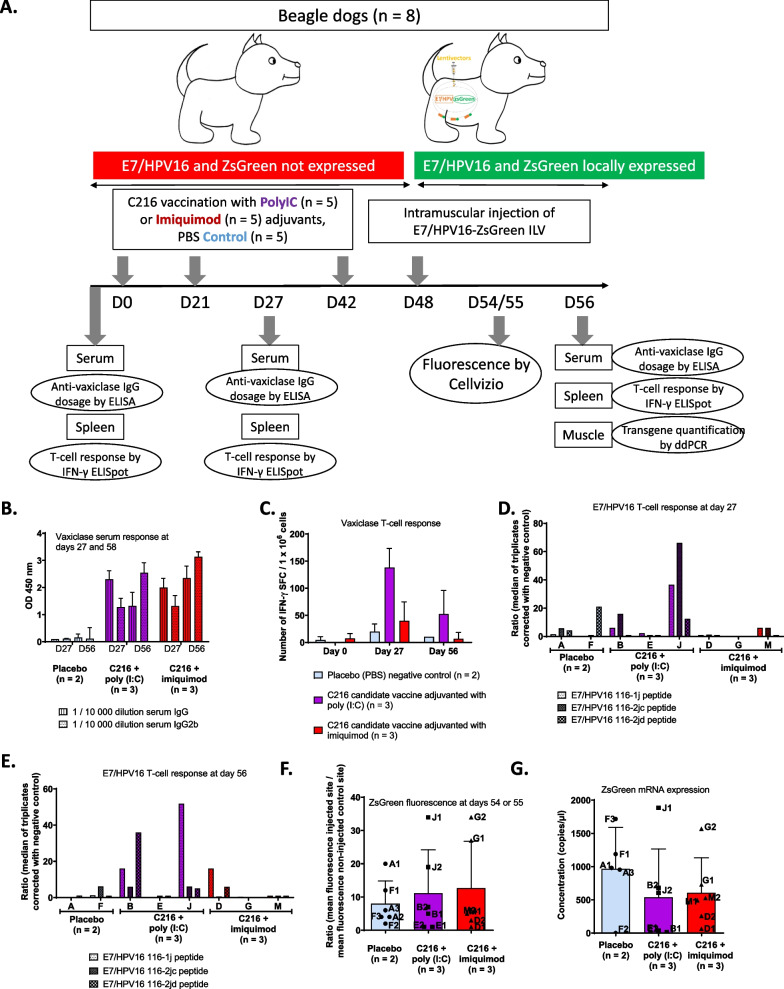
Evaluation of the efficacy of compound C216 as a prophylactic vaccine in dog. **A** Experimental design. **B** Total serum IgG and IgG2b were measured by ELISA, the optical density (OD) was obtained at 450 nm and the means of the ODs ± SD were calculated. **C**, **D**, and **E** T-cell stimulation was evaluated by IFN-γ ELISpot assay at days 27 and 56. Peripheral blood mononuclear cells were collected from dogs and stimulated in triplicate with Vaxiclase (**C**) or three pools of E7/HPV16 peptides (**D** and **E**). For each dog, the number of spot forming cells (SFC) corresponds to the median of triplicates and was corrected with the negative control (DMSO). The mean $\pm$ SD of the SFC/10^6^ cells was calculated for the three groups of vaccines. **D** and **E** For each E7/HPV16 peptide (116-1j, 116-2jc, and 116-2jd), instead of calculating the mean, the values for SFC/10^6^ cells at days 27 and 56 were divided to the value at day 0, to obtain a ratio for each dog. **F** The fluorescence was acquired with the Z probe at 488 nm using the Cellvizio imaging system and was corrected with that observed at the non-injected (NI) point, for every dog at days 54 or 55. The corrected fluorescence was calculated according to a ratio between the mean of the fluorescence intensity at the lentiviral point and the mean of the fluorescence intensity of the NI point. Finally, the means of the ratios ± SD were calculated. **G** ZsGreen mRNA expression was evaluated by RT-ddPCR for the three groups

Then, to test the efficacy of C216, E7/HPV16-ZsGreen ILV was injected IM at D48 (i.e. 6 days after the last vaccination) in dogs vaccinated with three consecutive intradermal injections of placebo (PBS) negative control (n = 2), C216 candidate vaccine adjuvanted with imiquimod (n = 3) or C216 candidate vaccine adjuvanted with Poly (I:C) (n = 3) (Fig. 4A). We first validated the immunogenicity of the C216 compound in dogs. The humoral immune response was evaluated by semi-quantification of total IgG and IgG2b synthesis one week after the second intradermal injection (D27) and at the end of the experiment, 8 days after the IM E7/HPV16-ZsGreen ILV injection (D56). Vaccinated dogs had a strong humoral immune response against Vaxiclase, characterized by IgG2b synthesis (Fig. 4B). We did not observe differences between dogs vaccinated with C216 candidate vaccine adjuvanted with imiquimod or with Poly (I:C). In the control group that was not injected with the C216 compound, only a low humoral immune response against Vaxiclase corresponding to the experimental background could be detected, as expected. Both C216 candidate vaccines adjuvanted with imiquimod or with Poly (I:C) also induced a cellular response against Vaxiclase characterized by IFN-γ synthesis (Fig. 4C) as previously shown in Totain et al. (submitted). In addition, the Vaxiclase specific response was still significant at the end of the experiment (D56) but only in dogs vaccinated with the vaccine adjuvanted with poly (I:C). These results confirmed that vaccinated dogs could develop a specific Th1 immune response against the candidate vaccine vector.

We then analyzed whether a cellular response specific to E7/HPV16 was also induced. As this response is dependent on the MHC haplotype of each dog, an individual evaluation was performed at D0 (to evaluate the specificity of E7/HPV16 IFN-γ ELISpot), after two consecutive vaccinations (D27, Fig. 4D) and after three consecutive vaccinations and E7/HPV16-ZsGreen ILV injection (D56, Fig. 4E). As expected, no specific response against the pools of peptides derived from E7/HPV16 could be detected in control dogs at D0, D27, and D56. A cellular immune response was detected in animals vaccinated with C216 adjuvanted with poly (I:C) at D27 in dog J and at D56 in dog J and dog B. Dog D vaccinated with C216 adjuvanted with poly (I:C) showed a moderate cellular response against one pool of peptides. As previously observed in Totain et al. (submitted), the induction of the E7/HPV16 T-cell response is variable in outbred beagle dogs.

To determine whether the presence of a cellular response could be associated with the in vivo elimination of cells expressing E7/HPV16, ZsGreen expression was monitored 6 (D54) or 7 (D55) days after ILV injection by Cellvizio measurement (Fig. 4F). The mean ratio of fluorescence expression between the three groups of dogs was not statistically different. When results were analyzed individually, we could not detect a relationship between the level of cellular response against E7/HPV16 and the extinction of fluorescence. For example, dog J presented the strongest E7/HPV16 specific T-cell response but also the highest level of ZsGreen fluorescence. By contrast, we were not able to detect a cellular response in dog E, whereas the fluorescence was almost abolished. The presence of E7/HPV16 expression was also confirmed by RT-ddPCR quantification of the E7/HP16-T2A-ZsGreen mRNA from the ILV injection site (Fig. 4G). There was no significant difference between the three groups. In addition, we observed a good correlation for each dog between the two methods of quantification of local E7/HPV16 expression.

In conclusion, despite good immunogenicity of the C216 compound, characterized by the induction of a specific Th-1 response, we were not able to detect any prophylactic efficacy in either mouse or dog models under our experimental conditions.

## Discussion

For several diseases that are highly specific to humans, animal models are lacking or inadequate. This is the case for HPV infections and associated neoplastic lesions. Indeed, HPVs have a very narrow host spectrum and do not infect classical preclinical models such as mouse or rat [15]. To circumvent this problem, several mouse models have been used. Existing genetically engineered mice have provided valuable information on the carcinogenic properties of HPV but are not relevant for testing potential vaccines [30].

The subcutaneous TC-1 cell tumor model is still the primary preclinical model for studying the prophylactic or therapeutic efficacy of HPV vaccine candidates [25, 31–36] or for the study of other potential therapeutic approaches [37]. TC-1 cells are modified C57BL/6 neoplastic mouse cells expressing E6 and E7 proteins from the HPV-16 serotype. After engraftment, it is possible to measure tumor development and assess the reduction of its size after treatment. Successful elimination of TC-1-induced tumors was observed with various vaccine candidates [25, 31–36]. Despite these results, no therapeutic vaccine against HPV has been proven to be effective in humans to date [38]. In particular, it is of note that ProCervix (GTL001) adjuvanted with Imiquimod is a vaccine candidate very similar to the C216 compound tested in this study. Instead of consisting of Vaxiclase carrying the three modified E7/HPV-16, E7/HPV-18, and E7/HPV-45 antigens, the two detoxified CyaAs from ProCervix only carry two non-oncogenic E7/HPV-16 or E7/HPV-18 antigens. Preclinical data on mice were very promising [11, 25], showing complete tumor regression with a TC-1 murine HPV16 E7-expressing tumor model [25]. However, ProCervix phase II final trial data showed no statistical differences in viral clearance rates between ProCervix and placebo groups. The lack of translation of these mouse preclinical data into efficient human therapy therefore brings into question the relevance of the TC-1 tumor model.

A significant immune response against the target molecule is also an important marker used during preclinical or phase I validation of a vaccine candidate. For example, the cell-mediated immune response is important for clearance of established infections and is therefore expected for a therapeutic vaccine [38]. In the phase I clinical trial of ProCervix, the induction of a cellular immune response in women was observed [3], confirming that detection of such an immune response is not sufficient for predicting the efficacy of a potential vaccine.

In this study, we used two very different models to evaluate C216 as a prophylactic vaccine.

As mentioned in the introduction, in the K14-E7/HPV16 mouse model, the oncogenic E7 protein is constitutively expressed in the skin [18], rendering these mice tolerant to the antigen and thus avoiding specific immune responses. In our E7^inv^ C57BL/6N transgenic mouse model, HPV16 expression is induced by local delivery of Cre recombinase via LentiFlash^®^ particles in a temporally and spatially controlled manner. GFP expression correlates with HPV16 expression as shown in Fig. 1. GFP expression was monitored for 35 days (data not shown), with a maximum expression between days 4 and 12 after Cre administration. This kinetic of analysis allowed us to choose day 5 for the first vaccination. As expected, the induction of HPV16 expression in these mice led to a strong immune response, as shown in Fig. 1E (IFN-y ELISpot assay). As a consequence, GFP reporter expression started to decrease at later time points, both in unvaccinated control mice and in vaccinated animals, making it impossible to quantify a specific effect of vaccination at late time points.

In dogs, the experimental design is voluntarily different in order to modify the immunological environment and to confirm our initial results in a different setting.

In these mouse and dog models, a specific E7/HPV16 T-cell response was observed. However, no reduction of E7/HPV16 levels was achieved. In contrast to the TC-1 tumor model, our results correlated with the results observed in phase I and II trials with ProCervix: despite the detection of a cellular response against E7/HPV16, there was a lack of efficacy of the candidate vaccine.

The mouse E7^inv^ C57BL/6N model allowed conditional expression of E7/HPV16 associated with eGFP fluorescence monitoring. eGFP was chosen because of its minimal immunogenicity in C57BL/6 mice [39]. By local Cre injection or crossing with a Cre or CreER^T2^ deleter mouse line, the expression of E7/HPV16 could be induced in any relevant cell type and at a chosen time. However, as this model was on an inbred background, it lacked the natural variation that is observed in human immune responses. Differences in innate immune responses between man and mouse, and maintenance in conventional laboratory conditions also reduce direct human translation [40–42]. However, the results of our study on the E7^inv^ model in beagle dogs strongly support the lack of efficacy of the C216 vaccine candidate. The canine immune system and immune responses are more similar to those of humans than to those of mouse, and can better reproduce inter-individual variations [22]. The dog model was also approved by regulatory authorities as the most suitable species in which to assess the safety of ProCervix. Moreover, the characterization of the local and systemic safety, as well as the immune response induced, in particular the cytotoxic T lymphocyte response, make dogs an additional relevant model for the preclinical evaluation necessary for the development of new vaccine candidates for human therapy. Indeed, here we observed a significant variation of the E7/HPV16 T cell response between dogs in contrast to what we found with the inbred E7^inv^ mouse model. These data were confirmed by another study, in which we demonstrated that the C216 vaccine candidate was immunogenic in dogs and induced a cellular response against E7, with variable intensity depending on the dog (Totain et al.submitted). As this study was conducted with a limited number of dogs, it is obviously not fully representative of the diversity that is observed in humans. However, these results were consistent with the phase I results with ProCervix, showing a specific cellular response non corelated to vaccine efficacy. Our previous results also indicated that the cellular response was stronger when the vaccine was associated with poly (I:C) than with imiquimod (Totain et al.submitted). These data were confirmed in this study, although the cellular response against HPV-16 E7 could not be detected in one dog that was vaccinated with C216 adjuvanted with poly (I:C), probably due to the dog leucocyte antigen haplotype of that animal.

One advantage of our dog model is that E7/HPV16 is expressed in the dogs’ cells using a lentiviral vector; therefore, E7 peptide DNA sequences are integrated into the cell genome and are further processed by the cells for MHC class I antigen presentation at the cell surface, mimicking a virus infection. Indeed, it has recently been demonstrated that lentiviral vectors are an efficient tool with which to mount in vivo specific CD8 + T cell responses when used as T-cell vaccines for direct expression of antigens [43].

Our two models also open the discussion on the evaluation of methods used in preclinical experiments and how to increase their translatability to the clinic. For example, TC-1 tumor preclinical experiments are used to predict the prophylactic or therapeutic efficacy of various HPV vaccine candidates but these data aren’t confirmed in human trials. For our mouse and dog models, to fully confirm their translation into human clinics, we would have needed to have an E7 vaccine whose effectiveness has been validated in humans. Currently, three prophylactic vaccines (Cervarix^®^, Gardasil^®^, and Gardasil^®^9) are effective at preventing human HPV infections [38]. However, as their target antigens are L1 capsid proteins, the models developed here are not suitable to investigate the vaccine effect and new ones should be developed that express the target antigens.

Another issue raised by our data is the impact of the unavailability of detailed records for ProCervix vaccine. Indeed, final detailed results of the Phase II trial are not publicly available. However, this Phase II clinical trial did not meet the primary endpoint of viral clearance in all infected patients 12 months after vaccination. In this Phase II study, there was heterogeneity among the patients recruited, with patients at different stages of the disease, from asymptomatic to ASCUS stage (https://clinicaltrials.gov/ct2/show/record/NCT01957878). Since detailed results of this Phase II study are not available, it is not known whether the vaccine was successful in any of the disease stage groups. This lack of data rends therefore difficult to make a direct comparison with the results of our study. Although the canine model is a very interesting step between mouse and human, our model can't mimic the complexity of the cervical papillomavirus with different phases of virus-induced cell transformation.

However, in our model, cells expressing E7 of HPV are not malignant. Thus, our model is probably close to the first steps of HPV evolution after infection in humans. We can assume that if the vaccine response induced in our study was not sufficient to eliminate non-malignant E7-expressing cells, these results would be a signal of weak efficacy in humans. Together with the previous point (validation of our model on an effective vaccine), this demonstrates the difficulty of defining relevant models to validate a drug.

However, these two points reinforce our conclusions regarding the importance of a design that tries to be as relevant as possible in animal models and that is not based solely on the use of so-called classical models (in this case TC-1 cells), whose validation may also be insufficient.

## Conclusions

We propose here new tools for the preclinical evaluation of vaccine candidates. This approach is easily transposable to the testing of other vaccine candidates. Even if these tools are not completely validated, and taking into consideration those that are currently available, which are not predictive of efficiency in humans, our approach to preclinical models paves the way to improved technical solutions, which are closer to the physiopathology of the targeted disease. After validation, these solutions could be a way to circumvent the lack of translation between preclinical data and clinical vaccine efficiency, at least in infectious diseases.

## Methods

### Animals

*Ethical statement*

All experimental procedures were performed in agreement with the EC directive 2010/63/UE86/609/CEE for the care and use of laboratory animals and every effort was made to minimize the number of animals used. This study was approved by the Local Ethical Committees (Com-Eth n°17 for mouse and Com-Eth n°86 for Dog) under the supervision of the French Ministry of education and research (APAFIS #9229–2,017,031,316,431,879 for the mouse study and #16,399–2,018,080,613,445,583 for the dog study) and accredited by the “Haut Conseil des Biotechnologies”(numbers #317 for the use of lentivectors and the expression of fluorescent proteins in dogs and #1811 for the use of lentivectors in dogs and mice and the expression of mutated E7-HPV).

#### Mouse line

Gt(ROSA)26Sor^tm1(CAG‐E7,‐EGFP)Ics^ knock-in heterozygous mice (abbreviated to E7^inv^/wt), which exhibit conditional expression of both E7/HPV16 non-oncogenic antigen and eGFP, were generated at the Institut Clinique de la Souris (Celphedia, Phenomin, ICS, Illkirch). This mouse model was engineered as detailed in Additional file 2. In brief, the final construct was linearized and electroporated in PHENOMIN-ICS proprietary derived C57BL/6N ES cells. Positive clones were selected by long-range PCR and further validated by Southern blot using both a Neo probe and a 3′ external probe (as described in [44]). A fully validated ES cell clone, which did not show any abnormalities by ddPCR (as described in [45]) and by Giesma karyotyping, was microinjected in BALB/cN blastocysts. Chimeras were obtained and germline transmission of the recombinant allele was achieved in a C57BL/6N pure genetic background. The Gt(ROSA)26Sor^tm1(CAG‐E7,‐EGFP)Ics^ line is archived in the Infrafrontier/EMMA mouse mutant resource [46].

The genotyping protocol is detailed in Additional file 3 and in [47].

C57BL/6N wild-type mice were purchased from Charles River Laboratory (Saint-Germain-Nuelles, France).

#### Dogs

Conventional female beagle dogs were purchased from a licensed kennel for animal breeding and husbandry (Centre d’élevage du domaine de souches, France). All dogs were dewormed and vaccinated against canine distemper virus, parvovirus, leptospirosis, infectious canine hepatitis, and parainfluenza virus. They were housed in an accredited facility in standard indoor and outdoor runs and were provided commercial dog food (Medium Adult, Royal Canin) and tap water ad libitum.

One dog aged 3 years on the day of the E7/HPV16‐ZsGreen integrating lentivirus (ILV) injection used in preliminary experiment and 8 dogs aged 8 months ± 1 month on the day of the first immunization were used. Dogs were acclimated for 3 weeks before experiments.

### Vaccine description, formulation and administration

#### Vaccine description

The C216 compound (Genticel) consisted of E7/HPV16, E7/HPV18 and E7/HPV45 recombined Vaxiclase^®^ (Genticel) protein. This antigen was prepared in 1 X PBS (Fisher BioReagents™) supplemented with urea (pH 7.4 ± 0.2). All animals received three injections intradermally on their back every three weeks (D0, D21, and D41 or 42). Two adjuvants were tested: polyinosinic:polycytidylic acid (poly (I:C)) VacciGrade^®^ (vac-pic, InvivoGen), or a topical 5% imiquimod cream (Aldara™). Imiquimod was applied immediately and 24 h after vaccination at injection sites by rubbing until complete absorption. The main difference between C216 and the first generation Procervix vaccine is an additional deletion of 93 AA in the catalytic domain of CyaA. This deletion allows the insertion of larger antigenic fragments, resulting in a greater diversity of CD4 and CD8 epitopes [24] and thus a better immune response. C216 is therefore expected to have better immunogenicity than the previous vector, ProCervix, used in the Phase II trial.

#### C216 construction and production

The DNA sequence of the adenylate cyclase of *B. pertussis*, CyaA (GeneBank: CAE41066.1) was optimized and synthesized (GeneCust) for expression in *Escherichia coli* as previously described [25], (Totain et al., submitted). A deletion of 93 amino acids from position 228 to position 320 of *B. pertussis* CyaA was performed, removing the calmodulin-interacting domain of CyaA [48] and thus inactivating its catalytic domain. This new recombinant CyaA was named Vaxiclase. To remove any oncogenic potential, the three E7 sequences (HVP16, HPV18, and HPV45) were each split into two fragments and the N-terminal portions of each were fused, followed by fusion of the three C-terminal portions. The acidic regions of E7 were also removed to avoid any deleterious effect of these domains [49, 50].

The plasmid was electroporated in the BLR bacterial strain (Novagen). The production of the recombinant Vaxiclase protein containing the E7/HVP16, E7/HPV18, and E7/HPV45 peptides (named C216) was induced by the addition of IPTG [51] after bacterial growth in classical medium. Purification of the expressed protein was achieved by chromatography procedures; ionic exchange affinity chromatography and hydrophobic exchange chromatography techniques were performed as described previously [26].

#### Formulation and administration in mice

Fifteen mice were randomly distributed across groups, with five mice per group, with the same distribution of females and males in each group. They were anesthetized under 2–2.5% isoflurane in O_2_ (Vetflurane^®^, Virbac) and vaccinated in each ear, either with an adjuvanted control (PBS with 1.83 M urea, 2.5 µg poly (I:C) and 25 mg imiquimod), or with C216 compound adjuvanted with poly (I:C) (10 µg C216, PBS with 1.83 M urea, 2.5 µg poly (I:C)) or with C216 compound adjuvanted with imiquimod (10 µg C216, PBS with 1.83 M urea, 25 mg imiquimod).

#### Formulation and administration in dogs

Eight dogs were randomly distributed into groups and were vaccinated on the back by intradermal injection, with control (PBS with 1.85 M urea, 150 µg poly (I:C) and 250 mg imiquimod) for 2 dogs, or C216 compound adjuvanted with poly (I:C) (600 µg C216, PBS with 1.85 M urea, 150 µg poly (I:C)) for 3 dogs, or with C216 compound adjuvanted with imiquimod (600 µg C216, PBS with 1.85 M urea, 250 mg imiquimod) for 3 dogs.

#### Criteria of selection of the antigen dose

For the mouse studies, we used the doses previously used in other mouse models (10 µg of Ag per mouse) for which vaccine efficacy (regression of TC-1 tumors) has been demonstrated [25]. For the dog formulation, we used the maximum dose used (600 µg of Ag per dog) to be as close as possible to the human phase I design [3].

### Integrative and non-integrative lentivectors

#### E7/HPV16‐ZsGreen integrative lentiviral vector preparation and quantification

Three plasmids were used to produce recombinant lentiviral particles [1]. The first plasmid, pLV gag-pol, provided a nucleic acid encoding the viral *gag* and *pol* genes, but lacking the *vif*, *vpr*, *vpu*, and *nef* genes. The second plasmid, pVSVG, provided a nucleic acid encoding the vesicular stomatitis virus envelope glycoprotein (VSV-G). A third self-inactivating expression plasmid encoded the immunogenic peptide E7 sequence from HPV16 described in [25]) under the control of the human elongation factor 1 alpha (EF1a) promoter and the ZsGreen fluorescent reporter protein, separated from the E7/HPV16 sequence by a T2A sequence. Viral vector production was performed in a 10-layer CellSTACK (Corning) after tri-transfection by standard calcium phosphate procedure of the three plasmids described above. Twenty-four hours after transfection, the supernatant was discarded and replaced by fresh medium, and the cells were incubated at 37 °C in a humidified atmosphere of 5% CO_2_ in air. After medium exchange, the supernatant was collected several times, and each harvest was clarified by centrifugation for 5 min at 3000 g before being microfiltered through a 0.45 µm pore size sterile filter unit (Stericup, Millipore). All supernatants were then pooled to supply the crude harvest. Concentration and purification were then performed on the crude harvest by ultrafiltration followed by diafiltration.

Transduction unit titration assays were performed as follows. HCT116 cells were seeded in a 96-well plate. Twenty-four hours later, five serial dilutions were performed with each vector sample and an rLV-EF1-GFP internal standard. Three days after transduction, cells were trypsinized and the titer (transducing units ml^−1^) was determined by qPCR after extraction of genomic DNA using the Nucleospin tissue gDNA extraction kit (Macherey–Nagel, Hoerdt, France). The titer, determined in transducing units per ml (TU ml^−1^) using qPCR, was normalized by an internal standard whose titer was previously determined by FACS.

#### *Cre LentiFlash*^*®*^* non-integrative vector preparation and quantification*

The LentiFlash^®^ system is a non-integrative lentiviral vector consisting of an RNA transfer method that exploits the bacteriophage MS2-Coat and its cognate 19-nt stem loop, as previously described [21]. Similar to the integrative lentiviral vectors’ process, three plasmids were used to produce recombinant LentiFlash^®^ particles. The first plasmid, pLV gag-pol, provided a nucleic acid encoding viral *gag* and *pol* genes, modified to bear the MS2-Coat within the *gag* gene. The pVSVG plasmid provided a nucleic acid encoding the vesicular stomatitis virus envelope glycoprotein (VSV-G). A third self-inactivating expression plasmid encoded the *Cre* recombinase gene, flanked by 12 repeats of the MS2 stem loops, to enable the mobilization of mRNA into lentiviral particles. The production, concentration, and purification processes were then the same as for the integrative particles.

LentiFlash^®^ physical particles were quantified by p24 ELISA assay. The p24 core antigen was detected directly on the viral supernatant with an HIV-1 p24 ELISA kit, according to manufacturer’s instructions (Perkin Elmer). The viral titer expressed in physical particles per ml was calculated from the amount of p24, knowing that 1 pg of p24 corresponds to 10^4^ physical particles.

#### Administration conditions

In both mice and dogs, lentivector injections were performed in the muscular fascia in the left posterior paw, after a slight skin incision. For each animal, a control injection was performed in the right posterior paw (Supplementary Table 1). All mice were injected with 20 µl of vector (LentiFlash^®^ Cre particles or E7/HPV16‐ZsGreen ILV) under intraperitoneal anesthesia with a mix of 60–70 mg kg^−1^ ketamine (Clorketam^®^ 1000, Vetoquinol) and 0.5 mg kg^−1^ medetomidine (Medetor^®^, Virbac) previously diluted in physiological serum. All dogs were injected with 50 µl lentivector (E7/HPV16‐ZsGreen ILV) under subcutaneous tranquilization with 0.01 mg kg^−1^ acepromazine (Calmivet^®^, Vetoquinol) diluted in physiological serum beforehand and 0.3 mg kg^−1^ butorphanol (Dolorex^®^, Merck). In addition, dogs received 6 mg of lidocaine (Lurocaine^®^ Vetoquinol), locally around the injected area, a few minutes before the lentivector administration.

The conditions of administration are described in Additional file 4: Table S1.

### Dog and mouse fluorescence acquisition

Before fluorescence acquisition, dogs and mice were sedated as described in 2.3.3. Fluorescence acquisition was performed in the muscular fascia at the paw, after a slight skin incision to trace the lentivector site. The detection of the local expression of the green fluorescence was realized by scanning the entire zone at 488 nm with the MiniZ or the Z probe from the Cellvizio system (Mauna Kea Technologies). For each animal, a 30–45 s video was captured for every injection area in addition to the control non-injected (NI) area (on the other paw). The data analysis was performed with IC-Viewer software (https://ic-viewer.software.informer.com/). The fluorescence intensity was calculated for each video by the mean ± SEM of the fluorescence intensity of all the frames. The mean ± SD was obtained every day, pooling animals according to groups.

### Blood and tissue sampling

#### Blood and serum

For dogs, blood samples were obtained by collection from the jugular vein into uncoated tubes and heparinized tubes. For mice, blood samplings were performed intracardially, under intraperitoneal overloaded anesthesia with a mix of ketamine and medetomidine diluted in physiological serum. Mice were then sacrificed by cervical dislocation. Sera were stored at − 80 °C until tested and uncoagulated blood was immediately used for isolation of peripheral blood mononuclear cells (PBMCs).

#### Muscle

A biopsy of the muscular fascia was sampled at each lentivector injection site, under anesthesia at the end of the fluorescence acquisition for dogs, and after sacrifice for mice. A piece of this sample was immersed in RNAlater RNA Stabilization Reagent^®^ (Qiagen) and stored at 4 °C.

#### Murine splenocytes

Spleens were collected aseptically and crushed in medium (RPMI 1640 Medium, GlutaMAX™ Supplement, Gibco™). The crushed spleen was filtered through a 100 µm cell strainer. The cell suspension was pelleted by centrifugation and red blood cell lysis was carried out with ACK (Ammonium-Chloride-Potassium) lysing buffer before being washed twice with the murine complete medium (RPMI complemented with 10% Eurobio heat-inactivated fetal bovine serum (FBS), 100 units-µg ml^−1^ penicillin/streptomycin (Gibco™) and 2.5 µg ml^−1^ amphotericin B (Sigma-Aldrich)).

#### Isolation of canine PBMCs

Whole blood from heparinized tubes was diluted in RPMI and PBMCs were isolated by density gradient centrifugation using Histopaque 1077 (density: 1.077 g ml^−1^; Sigma-Aldrich). After 3 washes and a 100 µm filtration, the resulting cells were suspended in the canine complete medium (RPMI containing 10% Eurobio heat-inactivated FBS, 100 units-µg/mL of penicillin/streptomycin (Gibco™) 1X sodium pyruvate (Gibco™), and 1X non-essential amino acids (Gibco™)).

For murine splenocytes and canine PBMCs, the concentration of viable cells was determined by microscopic examination using Gibco™ trypan blue exclusion. Viability was > 90% in all samples.

### Immune response analysis

#### Anti-vaxiclase IgG dosage by ELISA

The total levels of IgG and IgG2 antibodies specific for Vaxiclase^®^ (CyaA) were measured in mouse and dog sera. Nunc 96-well MaxiSorp plates were coated with 0.25 µg of Vaxiclase^®^ per well in carbonate buffer (pH 9.6) overnight at 4 °C. Between each step, plates were washed with PBT (Fisher BioReagent™ PBS containing 0.1% of Fisher BioReagent™ Tween™ 20) using the Wellwash™ microplate washer (Thermo Fisher Scientific). Non-specific sites were blocked with PBT/BSA (PBT containing 1% bovine serum albumin; Sigma-Aldrich) for 2 h at room temperature. Serial two-fold dilutions were tested, starting at 1:2 500 to 1:20 000 for mouse sera and 1:10 000 to 1:80 000 for dog sera, for 1 h of incubation at room temperature. The first dilution that did not saturate the ELISA in vaccinated animals was used to analyze the results (i.e. 1:5 000 for mice and 1:10 000 for dogs). Specific peroxidase-conjugated secondary antibodies were used: 0.04 µg per well of rabbit polyclonal anti-dog IgG (304–035-0030, Jackson Immuno Research), 0.02 µg per well of sheep polyclonal anti-dog IgG2 (A40-121P, Bethyl Laboratories), 0.1 µg per well of goat polyclonal anti-mouse IgG (0300-0108P, Bio-Rad) or 0.05 µg per well of goat polyclonal anti-mouse IgG2b (STAR134P, Bio-Rad) and were incubated 1 h at room temperature. Hybridization was carried out with a 15 min incubation with Sigma-Aldrich 3,3′,5,5′-Tetramethylbenzidine (TMB), and the reaction was stopped by adding 0.2 M H_2_SO_4_. Plates were read at 450 nm with µquant™ microplate spectrophotometer (BioTek^®^ Instruments).

#### T-cell response by IFN-γ ELISpot

T-cell responses were measured by ex vivo interferon-γ (IFN-γ) enzyme-linked immunospot (ELISpot) assay, following the manufacturer's recommendations (Mabtech). For murine splenocytes, the Mouse IFN-γ ELISpot BASIC (3321-2A) kit was used. For canine PBMCs, the horse IFN-gamma ELISpot BASIC (3117-2A) kit was used. In brief, 1 × 10^6^ splenocytes per well or 2 × 10^5^ PBMCs per well were stimulated in triplicate in 96-well MAIPSWU PVDF plates at 37 °C, 5% CO_2_ for 20–24 h. To do this, cells were cultured in murine or canine complete medium with 0.5% DMSO as a negative control or in the presence of 50 ng ml^−1^ phorbol 12-myristate 13-acetate plus 1 µM ionomycin calcium salt (Sigma-Aldrich) as a non-antigen-specific positive control. The antigen-specific response against the vaccine was tested with 0.5 µg per well Vaxiclase^®^ or 0.5 µg per well of each pool of peptides (Genticel). The following pools of peptides were derived from 15-mers, overlapping each other by 11 residues, covering the entire E7 sequence: HPV16 116-1j, HPV16 116-2jc, and HPV16 116-2jd. The number of spots was determined with the automated ImmunoSpot reader (CTL^®^). The median of the spot-forming cell (SFC) number was calculated between triplicates and converted for 1 × 10^6^ cells before being corrected by the negative control value for each animal. We calculated the mean ± SD of these values for each group of animals. To analyze the specific response of each HPV16 E7 peptide (116-1j, 116-2jc, and 116-2jd), instead of calculating the mean, the SFC values per 10^6^ cells at days 27 and 56 were compared to the values at day 0, obtaining a ratio, to eliminate the background response for each dog.

### ddPCR and RT-ddPCR

All primers and probes were designed following recommendations described in Lindner et al*.* 2021 [52]. Primer and probe sequences are provided in Additional file 5: Table S2. Sample extraction, RNA reverse transcription, droplet generation, PCR amplification, droplet quantification, and analysis are described in Lindner et al*.* 2020 [53]. Experiments were performed following dMIQE guidelines for reporting ddPCR experiments (Additional file 5: Table S2) [53, 54].

## Statistical analysis

Statistical analyses were performed using GraphPad Prism v9 software. Intergroup comparisons were performed using the Mann–Whitney test. The E7 protein-specific immune response following lentivector injections was determined using a one-tailed Wilcoxon statistical test. The significance threshold was set at p = 0.05.

## Supplementary Information

**Additional file 1**. **Fig. S1**: Detection of ZsGreen fluorescence from the E7/HPV16‐ZsGreen ILV with Cellvizio imaging system in muscle from wild-type mice. Mice were injected with the vector into the muscle at day 0.The detection of the local expression of green fluorescence was realized with the MiniZ probe using the Cellvizio® imaging system at 488 nm, from day 4 to day 13. A representative image was extracted from each video to visualize the fluorescence expression at the injected point in comparison with that at the non-injected point.The fluorescence intensity was expressed as the mean ± SEM of the fluorescence intensity of each injected site. Each mouse was tested every three days. Differences between groups were determined using the one-tailed Wilcoxon statistical test. SEM: standard error of the mean; ** p value < 0.01.

**Additional file 2**. Details of E7^inv^ mouse line generation.

**Additional file 3**. Genotyping protocol for E7^inv^ line.

**Additional file 4**. **Table S1**: Integrative and non-integrative lentivectors and administration conditions used in this study.

**Additional file 5**. **Table S2**: Details of ddPCR and RT-ddPCR experiments following dMIQE recommendations.

## Data Availability

Gt(ROSA)26Sor^tm1(CAG‐E7,‐EGFP)Ics^ knock-in heterozygous mice will be made available through the Infrafrontier European Mouse Mutant Archive—EMMA repository (https://www.infrafrontier.eu/emma/).

## Abbreviations

CyaA: Adenylate cyclase toxin
eGFP: Enhanced green fluorescent protein
ES: Embryonic stem
HPV: Human papillomavirus
ILV: Integrating lentivirus
IM: Intramuscular
NI: Non-injected
SD: Standard deviation
wt: Wild type
