# Supplementary figures and images for: Development of HPV16 mouse and dog models for more accurate prediction of human vaccine efficacy
Source: Lab Anim Res. 2023 Jun 12;39:14. doi: 10.1186/s42826-023-00166-3 (PMC10258489; doi:10.1186/s42826-023-00166-3)

## Slide 1
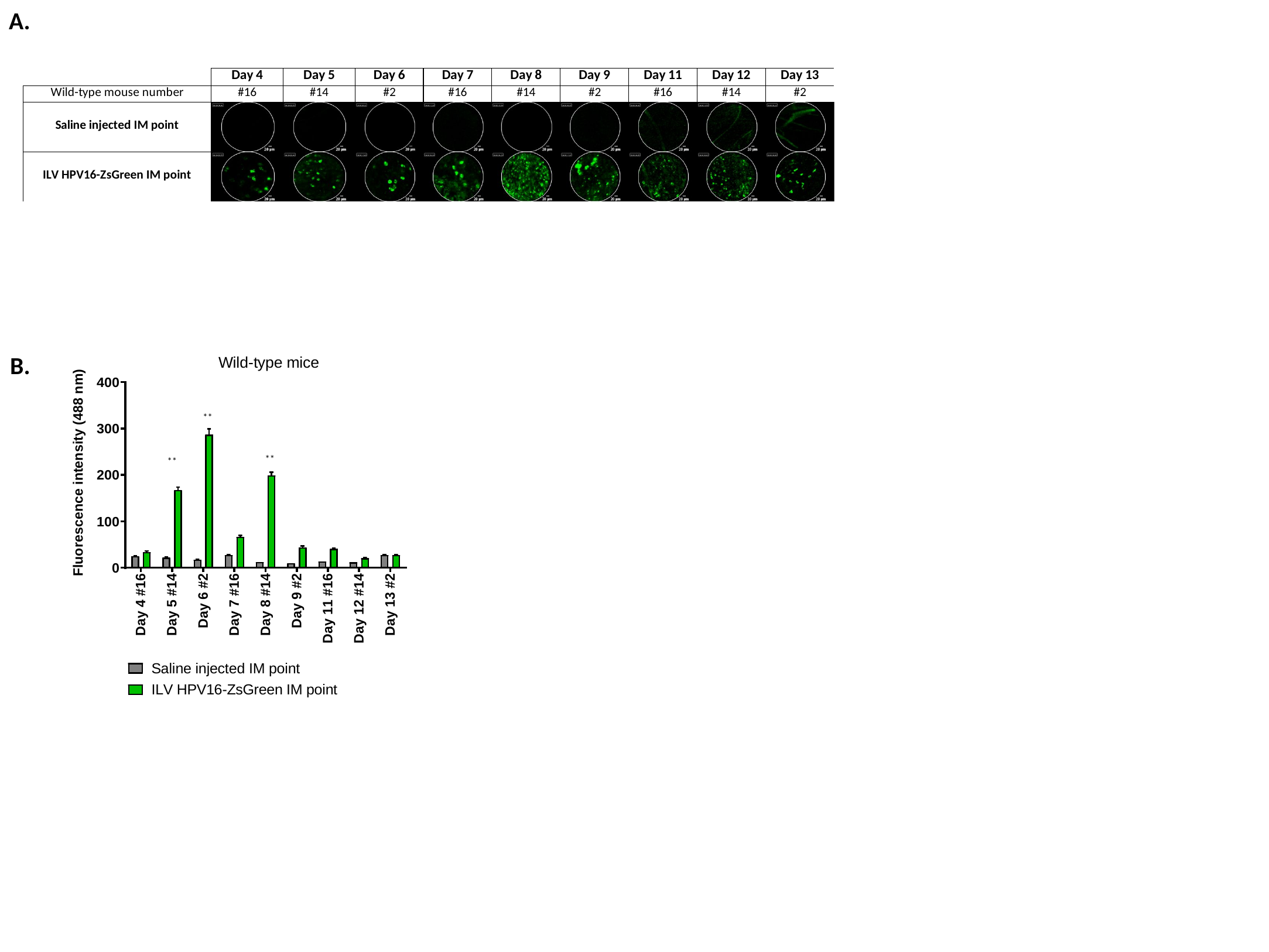

A.
**
**
**
B.

Supplement: Supplementary file 1 — Additional file 1. Fig. S1: Detection of ZsGreen fluorescence from the E7/HPV16‐ZsGreen ILV with Cellvizio imaging system in muscle from wild-type mice. Mice were injected with the vector into the muscle at day 0.The detection of the local expression of green fluorescence was realized with the MiniZ probe using the Cellvizio® imaging system at 488 nm, from day 4 to day 13. A representative image was extracted from each video to visualize the fluorescence expression at the injected point in comparison with that at the non-injected point.The fluorescence intensity was expressed as the mean ± SEM of the fluorescence intensity of each injected site. Each mouse was tested every three days. Differences between groups were determined using the one-tailed Wilcoxon statistical test. SEM: standard error of the mean; ** p value < 0.01. [file 42826_2023_166_MOESM1_ESM.pptx]
