# Supplementary material for: Development of HPV16 mouse and dog models for more accurate prediction of human vaccine efficacy
Source: Lab Anim Res. 2023 Jun 12;39:14. doi: 10.1186/s42826-023-00166-3 (PMC10258489; doi:10.1186/s42826-023-00166-3)
Supplement: Supplementary file 2 — Additional file 2. Details of E7inv mouse line generation. [file 42826_2023_166_MOESM2_ESM.docx]

**Supplementary File 1- detailed information on Gt(ROSA)26Sor^tm1(CAG‐E7,‐EGFP)Ics^ line generation**

# Targeting vector information

## Details of the E7/HPV16 - T2A - eGFP sequence used in the construct

In red: Kozak consensus sequence

In green: codon-optimized nucleotide sequence

In black: T2A sequence

In blue: SV40 polyA sequence

## Map and sequence of the targeting vector used for homologous recombination in ES cells


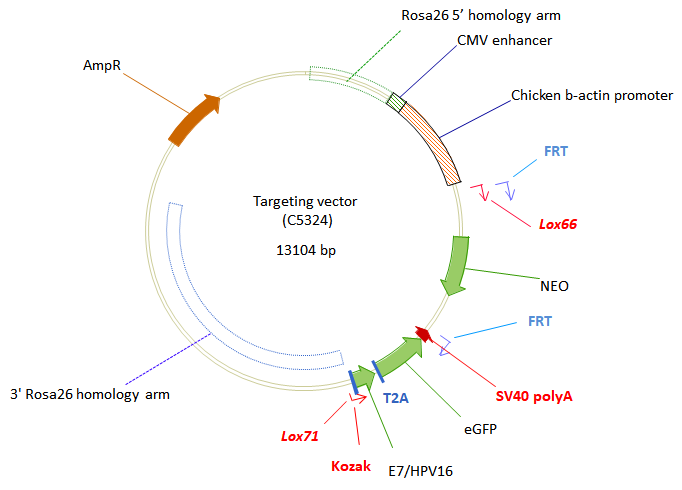


**DNA sequence of the targeting vector:**

attaaccctcactaaagggaacaaaagctggagctcgcggccgcggcgcgcccttggtgcgtttgcggggatgggcggccgcggcaggccctccgagcgtggtggagccgttctgtgagacagccgggtacgagtcgtgacgctggaaggggcaagcgggtggtgggcaggaatgcggtccgccctgcagcaaccggagggggagggagaagggagcggaaaagtctccaccggacgcggccatggctcgggggggggggggcagcggaggagcgcttccggccgacgtctcgtcgctgattggcttcttttcctcccgccgtgtgtgaaaacacaaatggcgtgttttggttggcgtaaggcgcctgtcagttaacggcagccggagtgcgcagccgccggcagcctcgctctgcccactgggtggggcgggaggtaggtggggtgaggcgagctggacgtgcgggcgcggtcggcctctggcggggcgggggaggggagggagggtcagcgaaagtagctcgcgcgcgagcggccgcccaccctccccttcctctgggggagtcgttttacccgccgccggccgggcctcgtcgtctgattggctctcggggcccagaaaactggcccttgccattggctcgtgttcgtgcaagttgagtccatccgccggccagcgggggcggcgaggaggcgctcccaggttccggccctcccctcggccccgcgccgcagagtctggccgcgcgcccctgcgcaacgtggcaggaagcgcgcgctgggggcggggacgggcagtagggctgagcggctgcggggcgggtgcaagcacgtttccgacttgagttgcctcaagaggggcgtgctgagccagacctccatcgcgcactccggggagtggagggaaggagcgagggctcagttgggctgttttggaggcaggaagcacttgctctcccaaagtcgctctgagttgttatcagtaagggagctgcagtggagtaggcggggagaaggccgcacccttctccggaggggggaggggagtgttgcaatacctttctgggagttctctgctgcctcctggcttctgaggaccgccctgggcctgggagaatcccttccccctcttccctcgtgatctgcaactccagtctttctggccggccgccaatagggactttccattgacgtcaatgggtggactatttacggtaaactgcccacttggcagtacatcaagtgtatcatatgccaagtacgccccctattgacgtcaatgacggtaaatggcccgcctggcattatgcccagtacatgaccttatgggactttcctacttggcagtacatctacgtattagtcatcgctattaccatgggtcgaggtgagccccacgttctgcttcactctccccatctcccccccctccccacccccaattttgtatttatttattttttaattattttgtgcagcgatgggggcggggggggggggggcgcgcgccaggcggggcggggcggggcgaggggcggggcggggcgaggcggagaggtgcggcggcagccaatcagagcggcgcgctccgaaagtttccttttatggcgaggcggcggcggcggcggccctataaaaagcgaagcgcgcggcgggcgggagtcgctgcgttgccttcgccccgtgccccgctccgcgccgcctcgcgccgcccgccccggctctgactgaccgcgttactcccacaggtgagcgggcgggacggcccttctcctccgggctgtaattagcgcttggtttaatgacggctcgtttcttttctgtggctgcgtgaaagccttaaagggctccgggagggccctttgtgcgggggggagcggctcggggggtgcgtgcgtgtgtgtgtgcgtggggagcgccgcgtgcggcccgcgctgcccggcggctgtgagcgctgcgggcgcggcgcggggctttgtgcgctccgcgtgtgcgcgaggggagcgcggccgggggcggtgccccgcggtgcgggggggctgcgaggggaacaaaggctgcgtgcggggtgtgtgcgtgggggggtgagcagggggtgtgggcgcggcggtcgggctgtaacccccccctgcacccccctccccgagttgctgagcacggcccggcttcgggtgcggggctccgtgcggggcgtggcgcggggctcgccgtgccgggcggggggtggcggcaggtgggggtgccgggcggggcggggccgcctcgggccggggagggctcgggggaggggcgcggcggccccggagcgccggcggctgtcgaggcgcggcgagccgcagccattgccttttatggtaatcgtgcgagagggcgcagggacttcctttgtcccaaatctggcggagccgaaatctgggaggcgccgccgcaccccctctagcgggcgcgggcgaagcggtgcggcgccggcaggaaggaaatgggcggggagggccttcgtgcgtcgccgcgccgccgtccccttctccatctccagcctcggggctgccgcagggggacggctgccttcgggggggacggggcagggcggggttcggcttctggcgtgtgaccggcggctctagagcctctgctaaccatgttcatgccttcttctttttcctacagctcctgggcaacgtgctggttattgtgctgtctcatcattttggcaaaggccggccataacttcgtatagcatacattatacgcccggtacggaaacgaagttcctattctctagaaagtataggaacttcgcggccaattctaccgggtaggggaggcgcttttcccaaggcagtctggagcatgcgctttagcagccccgctgggcacttggcgctacacaagtggcctctggcctcgcacacattccacatccaccggtaggcgccaaccggctccgttctttggtggccccttcgcgccaccttctactcctcccctagtcaggaagttcccccccgccccgcagctcgcgtcgtgcaggacgtgacaaatggaagtagcacgtctcactagtctcgtgcagatggacagcaccgctgagcaatggaagcgggtaggcctttggggcagcggccaatagcagctttgctccttcgctttctgggctcagaggctgggaaggggtgggtccgggggcgggctcaggggcgggctcaggggcggggcgggcgcccgaaggtcctccggaggcccggcattctgcacgcttcaaaagcgcacgtctgccgcgctgttctcctcttcctcatctccgggcctttcgacctgcagccaatatgggatcggccattgaacaagatggattgcacgcaggttctccggccgcttgggtggagaggctattcggctatgactgggcacaacagacaatcggctgctctgatgccgccgtgttccggctgtcagcgcaggggcgcccggttctttttgtcaagaccgacctgtccggtgccctgaatgaactgcaggacgaggcagcgcggctatcgtggctggccacgacgggcgttccttgcgcagctgtgctcgacgttgtcactgaagcgggaagggactggctgctattgggcgaagtgccggggcaggatctcctgtcatctcaccttgctcctgccgagaaagtatccatcatggctgatgcaatgcggcggctgcatacgcttgatccggctacctgcccattcgaccaccaagcgaaacatcgcatcgagcgagcacgtactcggatggaagccggtcttgtcgatcaggatgatctggacgaagagcatcaggggctcgcgccagccgaactgttcgccaggctcaaggcgcgcatgcccgacggcgaggatctcgtcgtgacccatggcgatgcctgcttgccgaatatcatggtggaaaatggccgcttttctggattcatcgactgtggccggctgggtgtggcggaccgctatcaggacatagcgttggctacccgtgatattgctgaagagcttggcggcgaatgggctgaccgcttcctcgtgctttacggtatcgccgctcccgattcgcagcgcatcgccttctatcgccttcttgacgagttcttctgaggggatccgctgtaagtctgcagaaattgatgatctattaaacaataaagatgtccactaaaatggaagtttttcctgtcatactttgttaagaagggtgagaacagagtacctacattttgaatggaaggattggagctacgggggtgggggtggggtgggattagataaatgcctgctctttactgaaggctctttactattgctttatgataatgtttcatagttggatatcataatttaaacaagcaaaaccaaattaagggccagctcattcctcccactcatgatctatagatctatagatctctcgtgggatcattgtttttctcttgattcccactttgtggttctaagtactgtggtttccaaatgtgtcagtttcatagcctgaagaacgagatcagcagcctctgttccacatacacttcattctcagtattgttttgccaagttctaattccatcagaagctcgataccgtcgaggaagttcctattctctagaaagtataggaacttcccgcggatccatcgaccccctgcaggccagacatgataagatacattgatgagtttggacaaaccacaactagaatgcagtgaaaaaaatgctttatttgtgaaatttgtgatgctattgctttatttgtaaccattataagctgcaataaacaagttcattacttgtacagctcgtccatgccgagagtgatcccggcggcggtcacgaactccagcaggaccatgtgatcgcgcttctcgttggggtctttgctcagggcggactgggtgctcaggtagtggttgtcgggcagcagcacggggccgtcgccgatgggggtgttctgctggtagtggtcggcgagctgcacgctgccgtcctcgatgttgtggcggatcttgaagttcaccttgatgccgttcttctgcttgtcggccatgatatagacgttgtggctgttgtagttgtactccagcttgtgccccaggatgttgccgtcctccttgaagtcgatgcccttcagctcgatgcggttcaccagggtgtcgccctcgaacttcacctcggcgcgggtcttgtagttgccgtcgtccttgaagaagatggtgcgctcctggacgtagccttcgggcatggcggacttgaagaagtcgtgctgcttcatgtggtcggggtagcggctgaagcactgcacgccgtaggtcagggtggtcacgagggtgggccagggcacgggcagcttgccggtggtgcagatgaacttcagggtcagcttgccgtaggtggcatcgccctcgccctcgccggacacgctgaacttgtggccgtttacgtcgccgtccagctcgaccaggatgggcaccaccccggtgaacagctcctcgcccttgctcaccatagggccgggattctcctccacgtcaccgcatgttagaagacttcctctgccctcttcctcgctgctgtcgttcagctgctcgtagcagtacaggtcggttgtctcgggctgcaggtccagcatgtactcgtgcagggtgggggtgtcgccgtgcatgggcttctggctgcagatggggcacacgatgcccagggtgcccatcagcagatcttccagggttctgatgtccacgtgggtgctctgcacgcacagtctcagggtgctgtcgcacttgcagcagaatgtcacgatgttgtagtgggctctatcaggttcggcctgtccggcagggccgtcgatctcgtcctccatggtggcggctataacttcgtataatgtatgctatacgaacggtaggccactgaggccgaagatgggcgggagtcttctgggcaggcttaaaggctaacctggtgtgtgggcgttgtccttaaggggaattgaacaggtgtaaaattggagggacaagacttcccacagattttcggttttgtcgggaagttttttaataggggcaaataaggaaaatgggaggataggtagtcatctggggttttatgcagcaaaactacaggttattattgcttgtgatccgcctcggagtattttccatcgaggtagattaaagacatgctcacccgagttttatactctcctgcttgagatccttactacagtatgaaattacagtgtcgcgagttagactatgtaagcagaattttaatcatttttaaagagcccagtacttcatatccatttctcccgctccttctgcagccttatcaaaaggtattttagaacactcattttagccccattttcatttattatactggcttatccaacccctagacagagcattggcattttccctttcctgatcttagaagtctgatgactcatgaaaccagacagattagttacatacaccacaaatcgaggctgtagctggggcctcaacactgcagttcttttataactccttagtacactttttgttgatcctttgccttgatccttaattttcagtgtctatcacctctcccgtcaggtggtgttccacatttgggcctattctcagtccagggagttttacaacaatagatgtattgagaatccaacctaaagcttaactttccactcccatgaatgcctctctcctttttctccatttataaactgagctattaaccattaatggtttccaggtggatgtctcctcccccaatattacctgatgtatcttacatattgccaggctgatattttaagacattaaaaggtatatttcattattgagccacatggtattgattactgcttactaaaattttgtcattgtacacatctgtaaaaggtggttccttttggaatgcaaagttcaggtgtttgttgtctttcctgacctaaggtcttgtgagcttgtattttttctatttaagcagtgctttctcttggactggcttgactcatggcattctacacgttattgctggtctaaatgtgattttgccaagcttcttcaggacctataattttgcttgacttgtagccaaacacaagtaaaatgattaagcaacaaatgtatttgtgaagcttggtttttaggttgttgtgttgtgtgtgcttgtgctctataataatactatccaggggctggagaggtggctcggagttcaagagcacagactgctcttccagaagtcctgagttcaattcccagcaaccacatggtggctcacaaccatctgtaatgggatctgatgccctcttctggtgtgtctgaagaccacaagtgtattcacattaaataaataaatcctccttcttcttcttttttttttttttaaagagaatactgtctccagtagaatttactgaagtaatgaaatactttgtgtttgttccaatatggtagccaataatcaaattactctttaagcactggaaatgttaccaaggaactaatttttatttgaagtgtaactgtggacagaggagccataactgcagacttgtgggatacagaagaccaatgcagactttaatgtcttttctcttacactaagcaataaagaaataaaaattgaacttctagtatcctatttgtttaaactgctagctttacttaacttttgtgcttcatctatacaaagctgaaagctaagtctgcagccattactaaacatgaaagcaagtaatgataattttggatttcaaaaatgtagggccagagtttagccagccagtggtggtgcttgcctttatgcctttaatcccagcactctggaggcagagacaggcagatctctgagtttgagcccagcctggtctacacatcaagttctatctaggatagccaggaatacacacagaaaccctgttggggaggggggctctgagatttcataaaattataattgaagcattccctaatgagccactatggatgtggctaaatccgtctacctttctgatgagatttgggtattattttttctgtctctgctgttggttgggtcttttgacactgtgggctttctttaaagcctccttcctgccatgtggtctcttgtttgctactaacttcccatggcttaaatggcatggctttttgccttctaagggcagctgctgagatttgcagcctgatttccagggtggggttgggaaatctttcaaacactaaaattgtcctttaattttttttttaaaaaatgggttatataataaacctcataaaatagttatgaggagtgaggtggactaatattaaatgagtccctcccctataaaagagctattaaggctttttgtcttatacttaactttttttttaaatgtggtatctttagaaccaagggtcttagagttttagtatacagaaactgttgcatcgcttaatcagattttctagtttcaaatccagagaatccaaattcttcacagccaaagtcaaattaagaatttctgacttttaatgttaatttgcttactgtgaatataaaaatgatagcttttcctgaggcagggtctcactatgtatctctgcctgatctgcaacaagatatgtagactaaagttctgcctgcttttgtctcctgaatactaaggttaaaatgtagtaatacttttggaacttgcaggtcagattcttttataggggacacactaagggagcttgggtgatagttggtaaaatgtgtttcaagtgatgaaaacttgaattattatcaccgcaacctactttttaaaaaaaaaagccaggcctgttagagcatgcttaagggatccctaggacttgctgagcacacaagagtagttacttggcaggctcctggtgagagcatatttcaaaaaacaaggcagacaaccaagaaactacagttaaggttacctgtctttaaaccatctgcatatacacagggatattaaaatattccaaataatatttcattcaagttttcccccatcaaattgggacatggatttctccggtgaataggcagagttggaaactaaacaaatgttggttttgtgatttgtgaaattgttttcaagtgatagttaaagcccatgagatacagaacaaagctgctatttcgaggtctcttggtttatactcagaagcacttctttgggtttccctgcactatcctgatcatgtgctaggcctaccttaggctgattgttgttcaaataaacttaagtttcctgtcaggtgatgtcatatgatttcatatatcaaggcaaaacatgttatatatgttaaacatttgtacttaatgtgaaagttaggtctttgtgggtttgatttttaattttcaaaacctgagctaaataagtcatttttacatgtcttacatttggtggaattgtataattgtggtttgcaggcaagactctctgacctagtaaccctacctatagagcactttgctgggtcacaagtctaggagtcaagcatttcaccttgaagttgagacgttttgttagtgtatactagtttatatgttggaggacatgtttatccagaagatattcaggactatttttgactgggctaaggaattgattctgattagcactgttagtgagcattgagtggcctttaggcttgaattggagtcacttgtatatctcaaataatgctggccttttttaaaaagcccttgttctttatcaccctgttttctacataatttttgttcaaagaaatacttgtttggatctccttttgacaacaatagcatgttttcaagccatattttttttcctttttttttttttttggtttttcgagacagggtttctctgtatagccctggctgtcctggaactcactttgtagaccaggctggcctcgaactcagaaatccgcctgcctctgcctcctgagtgccgggattaaaggcgtgcaccaccacgcctggctaagttggatattttgttatataactataaccaatactaactccactgggtggatttttaattcagtcagtagtcttaagtggtctttattggcccttcattaaaatctactgttcactctaacagaggctgttggtactagtggcacttaagcaacttcctacggatatactagcagattaagggtcagggatagaaactagtctagcgttttgtatacctaccagctttatactaccttgttctgatagtcgacctcgagggggggcccggtacccaattcgccctatagtgagtcgtattacgcgcgctcactggccgtcgttttacaacgtcgtgactgggaaaaccctggcgttacccaacttaatcgccttgcagcacatccccctttcgccagctggcgtaatagcgaagaggcccgcaccgatcgcccttcccaacagttgcgcagcctgaatggcgaatgggacgcgccctgtagcggcgcattaagcgcggcgggtgtggtggttacgcgcagcgtgaccgctacacttgccagcgccctagcgcccgctcctttcgctttcttcccttcctttctcgccacgttcgccggctttccccgtcaagctctaaatcgggggctccctttagggttccgatttagtgctttacggcacctcgaccccaaaaaacttgattagggtgatggttcacgtagtgggccatcgccctgatagacggtttttcgccctttgacgttggagtccacgttctttaatagtggactcttgttccaaactggaacaacactcaaccctatctcggtctattcttttgatttataagggattttgccgatttcggcctattggttaaaaaatgagctgatttaacaaaaatttaacgcgaattttaacaaaatattaacgcttacaatttaggtggcacttttcggggaaatgtgcgcggaacccctatttgtttatttttctaaatacattcaaatatgtatccgctcatgagacaataaccctgataaatgcttcaataatattgaaaaaggaagagtatgagtattcaacatttccgtgtcgcccttattcccttttttgcggcattttgccttcctgtttttgctcacccagaaacgctggtgaaagtaaaagatgctgaagatcagttgggtgcacgagtgggttacatcgaactggatctcaacagcggtaagatccttgagagttttcgccccgaagaacgttttccaatgatgagcacttttaaagttctgctatgtggcgcggtattatcccgtattgacgccgggcaagagcaactcggtcgccgcatacactattctcagaatgacttggttgagtactcaccagtcacagaaaagcatcttacggatggcatgacagtaagagaattatgcagtgctgccataaccatgagtgataacactgcggccaacttacttctgacaacgatcggaggaccgaaggagctaaccgcttttttgcacaacatgggggatcatgtaactcgccttgatcgttgggaaccggagctgaatgaagccataccaaacgacgagcgtgacaccacgatgcctgtagcaatggcaacaacgttgcgcaaactattaactggcgaactacttactctagcttcccggcaacaattaatagactggatggaggcggataaagttgcaggaccacttctgcgctcggcccttccggctggctggtttattgctgataaatctggagccggtgagcgtgggtctcgcggtatcattgcagcactggggccagatggtaagccctcccgtatcgtagttatctacacgacggggagtcaggcaactatggatgaacgaaatagacagatcgctgagataggtgcctcactgattaagcattggtaactgtcagaccaagtttactcatatatactttagattgatttaaaacttcatttttaatttaaaaggatctaggtgaagatcctttttgataatctcatgaccaaaatcccttaacgtgagttttcgttccactgagcgtcagaccccgtagaaaagatcaaaggatcttcttgagatcctttttttctgcgcgtaatctgctgcttgcaaacaaaaaaaccaccgctaccagcggtggtttgtttgccggatcaagagctaccaactctttttccgaaggtaactggcttcagcagagcgcagataccaaatactgtccttctagtgtagccgtagttaggccaccacttcaagaactctgtagcaccgcctacatacctcgctctgctaatcctgttaccagtggctgctgccagtggcgataagtcgtgtcttaccgggttggactcaagacgatagttaccggataaggcgcagcggtcgggctgaacggggggttcgtgcacacagcccagcttggagcgaacgacctacaccgaactgagatacctacagcgtgagctatgagaaagcgccacgcttcccgaagggagaaaggcggacaggtatccggtaagcggcagggtcggaacaggagagcgcacgagggagcttccagggggaaacgcctggtatctttatagtcctgtcgggtttcgccacctctgacttgagcgtcgatttttgtgatgctcgtcaggggggcggagcctatggaaaaacgccagcaacgcggcctttttacggttcctggccttttgctggccttttgctcacatgttctttcctgcgttatcccctgattctgtggataaccgtattaccgcctttgagtgagctgataccgctcgccgcagccgaacgaccgagcgcagcgagtcagtgagcgaggaagcggaagagcgcccaatacgcaaaccgcctctccccgcgcgttggccgattcattaatgcagctggcacgacaggtttcccgactggaaagcgggcagtgagcgcaacgcaattaatgtgagttagctcactcattaggcaccccaggctttacactttatgcttccggctcgtatgttgtgtggaattgtgagcggataacaatttcacacaggaaacagctatgaccatgattacgccaagcgcgca

# Screening of ES recombinant clones

The targeting vector was electroporated in the proprietary C57BL/6NCrl S4 cell line.

Transfected ES clones were submitted to neomycin selection (G418) and 93 resistant ES clones were isolated. The clones were then submitted to the screening process allowing secured identification of those harboring the expected recombination events at both ends of the targeting vector.

The screening process steps are:

Step 1: Identification of candidate recombinant clones by initial 5’ long-range PCR

Step 2: A dozen of 5’ PCR positive clones are confirmed for both 5’ and 3’ recombination events by long-range PCR

Step 3: Positive clones in step 2 are further validated by Southern blot analysis using internal and external probes

Step 4: The karyotype of a validated clone is verified using ddPCR aneuploidy screening and Giemsa staining

## 5’ and 3’ long-range PCR screening strategy


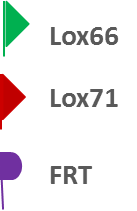

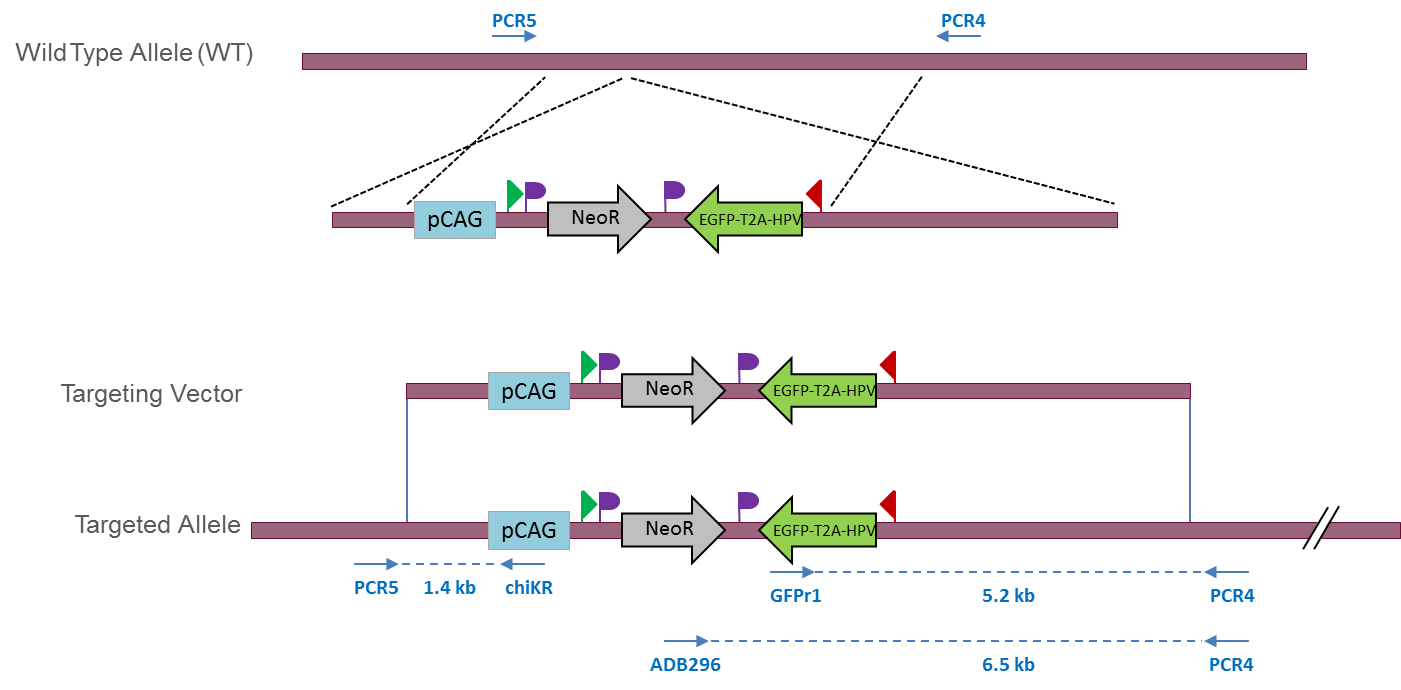


| PCR | Primer Name | Primer sequences | PCR product size |
| --- | --- | --- | --- |
| 5' PCR | PCR5 | GGTAGGGGATCGGGACTCTGGCGGG | 1.4 kb |
|  | chiKR | GGAGAGTGAAGCAGAACGTGGGGCT |  |
| 3’ PCR | ADB296 | AGGGGCTCGCGCCAGCCGAACTGTT | 6.5 kb |
|  | PCR4 | CTCAGTGGCTCAACAACACTTGGTC |  |
| 3’ PCR | GFPr1 | CCTTCAGCTCGATGCGGTTCACCAG | 5.2 kb |
|  | PCR4 | CTCAGTGGCTCAACAACACTTGGTC |  |

## Confirmation and validation of candidate recombinant ES clones by 5’ and 3’ PCR (step 2)


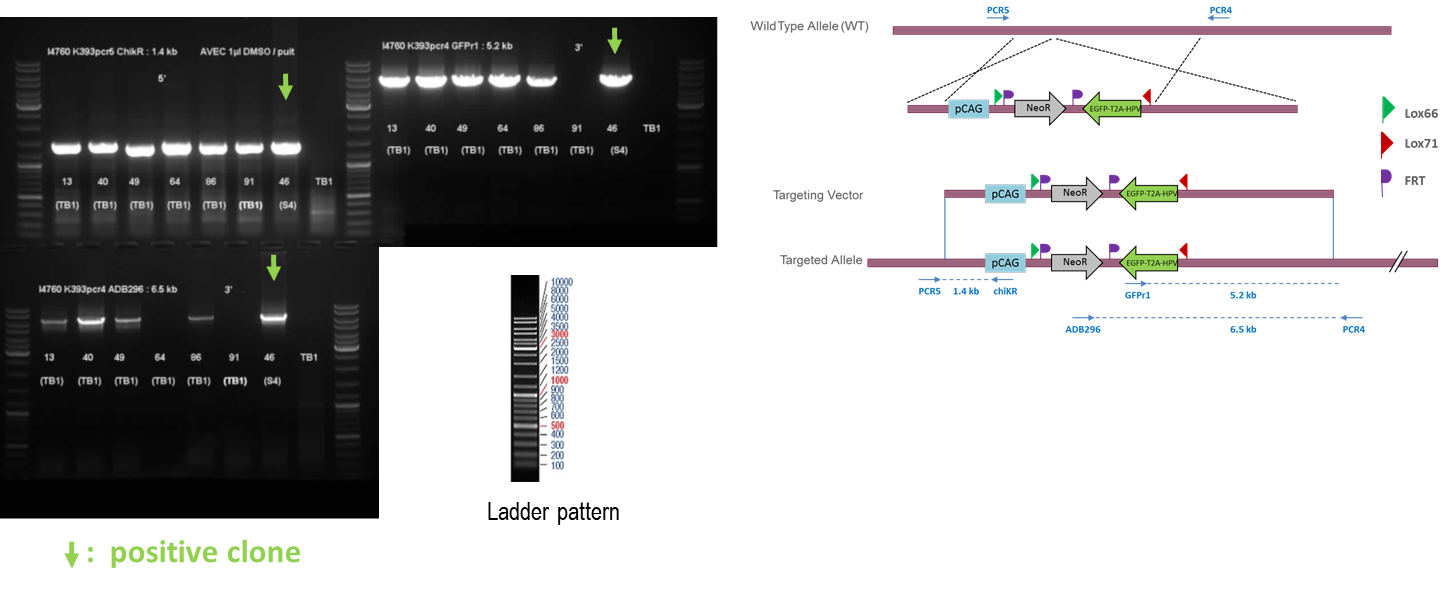


One candidate clone in the S4 ES cell line was identified by 5’ PCR screening and was further analyzed by 5’ and 3’ PCRs screening. This clone (clone #46) was confirmed after amplification.

## Recombinant ES clones validation by Southern blot using a Neo internal probe (step 3a)

Southern blot - Neo 5’

**BstEII 9.5**

**EcoNI 8.6**

Southern blot - Neo 3’

**Schematic Southern blot validation strategy**

**PacI 12.7**

15-

10-

8-

6-

**ApaI 11.7**

**13 40 49 86 46 13 40 49 86 46**

**13 40 49 86 46 13 40 49 86 46**

**: positive clone**

15-

10-

8-

6-


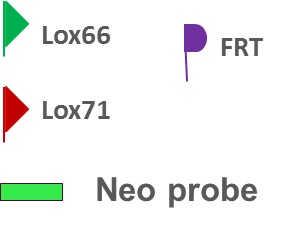

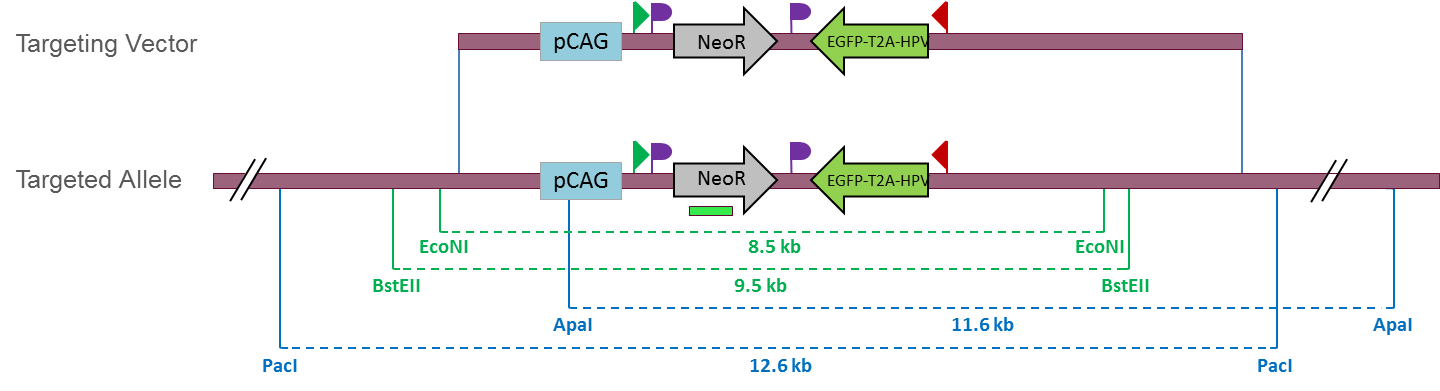


**Sequence of Neo probe**

AGAAGAACTCGTCAAGAAGGCGATAGAAGGCGATGCGCTGCGAATCGGGAGCGGCGATACCGTAAAGCACGAGGAAGCGGTCAGCCCATTCGCCGCCAAGCTCTTCAGCAATATCACGGGTAGCCAACGCTATGTCCTGATAGCGGTCCGCCACACCCAGCCGGCCACAGTCGATGAATCCAGAAAAGCGGCCATTTTCCACCATGATATTCGGCAAGCAGGCATCGCCATGGGTCACGACGAGATCCTCGCCGTCGGGCATGCGCGCCTTGAGCCTGGCGAACAGTTCGGCTGGCGCGAGCCCCTGATGCTCTTCGTCCAGATCATCCTGATCGACAAGACCGGCTTCCATCCGAGTACGTGCTCGCTCGATGCGATGTTTCGCTTGGTGGTCGAATGGGCAGGTAGCCGGATCAAGCGTATGCAGCCGCCGCATTGCATCAGCCATGATGGATACTTTCTCGGCAGGAGCAAGGTGAGATGACAGGAGATCCTGCCCCGGCACTTCGCCCAATAGCAGCCAGTCCCTTCCCGCTTCAGTGACAACGTCGAGCACAGCTGCGCAAGGAACGCCCGTCGTGGCCAGCCACGATAGCCGCGCTGCCTCGTCCTGCAG

**Digestions used to validate the 5’ and 3’ insertion**

| Probe | Name | Genomic DNA digest | Targeted Allele (kb) |
| --- | --- | --- | --- |
| Neo | 5’ digests | BstEII | 11.6 |
|  |  | EcoNI | 9.5 |
|  | 3’ digests | ApaI | 11.6 |
|  |  | PacI | 12.7 |

## Recombinant ES clones validation by Southern blot using an external probe (step 3b)


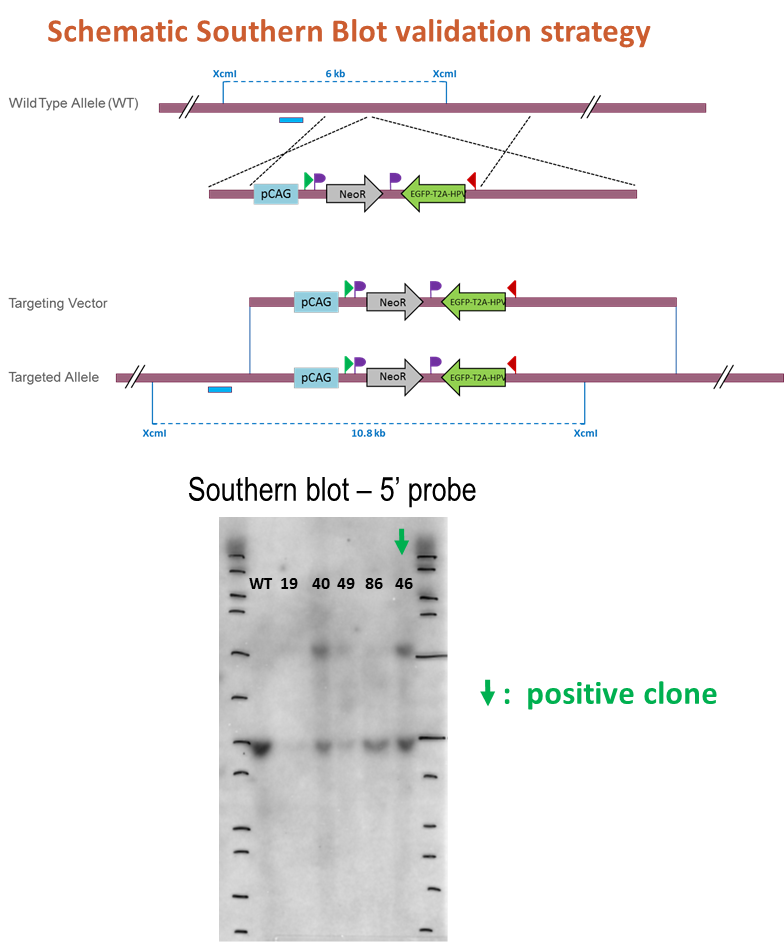


**Sequence of 5’ probe**

TATGTGTATTTTGAGAGCAGGGTTGGGAGGCCTCTCCTGAAAAGGGTATAAACGTGGAGTAGGCAATACCCAGGCAAAAAGGGGAGACCAGAGTAGGGGGAGGGGAAGAGTCCTGACCCAGGGAAGACATTAAAAAGGTAGTGGGGTCGACTAGATGAAGGAGAGCCTTTCTCTCTGGGCAAGAGCGGTGCAATGGTGTGTAAAGGTAGCTGAGAAGACGAAAAGGGCAAGCATCTTCCTGCTACCAGGCTGGGGAGGCCCAGGCCCACGACCCCGAGGAGAGGGAACGCAGGGAGACTGAGGTGACCCTTCTTTCCCCCGGGGCCCGGTCGTGTGGTTCGGTGTCTCTTTTCTGTTGGACCCTTACCTTGACCCAGGC

**Digestions used to validate the 5’ and 3’ insertion**

| Probe | Name | Genomic DNA digest | WT allele (kb) | Targeted Allele (kb) |
| --- | --- | --- | --- | --- |
| 5’ external probe | 5’ digest | XcmI | 6 | 10.8 |

## Karyotyping (step 4)

Clone #46 was karyotyped by ddPCR as described in Codner *et al.* (2016) and by Giemsa metaphase staining. Results of aneuploidy analysis are presented in the table below.

| Clone ID | ddPCR | Giemsa |
| --- | --- | --- |
| 46 | Pass | Pass |

**Reference:** Codner, G.F., Lindner, L., Caulder, A., Wattenhofer-Donzé, M., Radage, A., Mertz, A., Eisenmann, B., Mianné, J., Evans, E.P., Beechey, C.V., Fray, M.D., Birling, M.-C., Hérault, Y., Pavlovic, G., Teboul, L Aneuploidy screening of embryonic stem cell clones by metaphase karyotyping and droplet digital polymerase chain reaction. BMC Cell Biology 2016 doi:10.1186/s12860-016-0108-6

## Additional control of mutant ES cells

Cre-mediated inversion (E7^inv^ conversion to E7^+^) was verified at the ES cell level and the absence of any leakiness in the expression of the E7/HPV16 mRNA was checked by RT-qPCR (data not shown) and by fluorescence evaluation.

## Verification of Cre-mediated inversion efficiency (E7inv conversion to E7+)

Lox66 and Lox71 were used in the targeting construct (see Oberdoerffer *et al.* 2003). These Lox sites allow mainly a unidirectional inversion of the HPV-T2A-eGFP fragment at a specific time/cell in the mouse when the Cre is expressed.


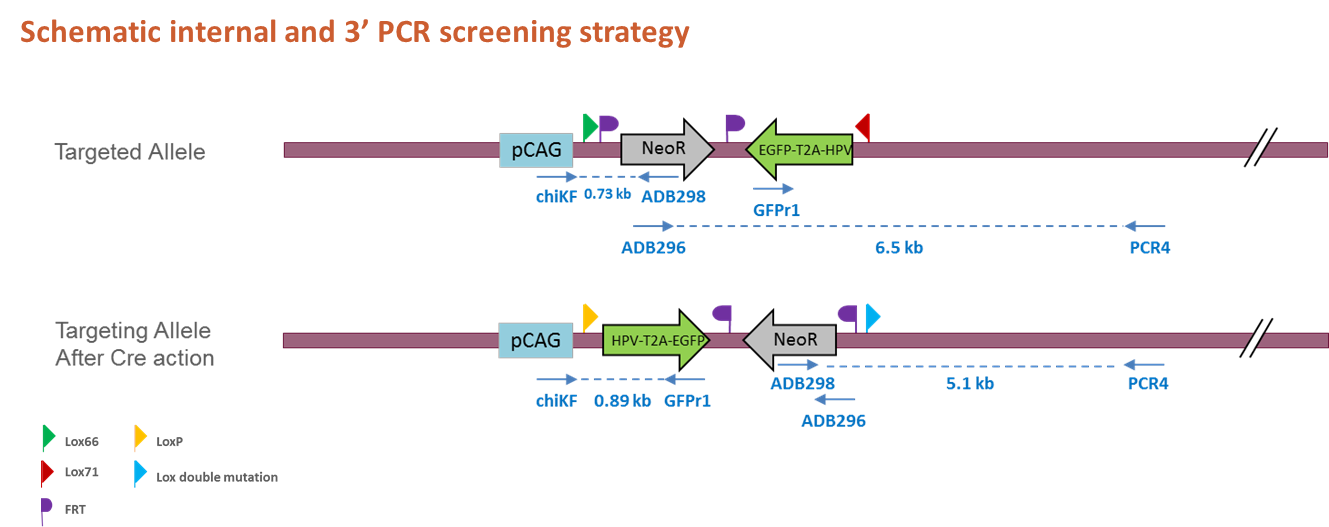


| PCR | Primer Name | Primer sequences | PCR product size |
| --- | --- | --- | --- |
| Internal PCR | chiKF | GGCTCTAGAGCCTCTGCTAACCATG | 0.73 kb |
|  | ADB298 | GCGGCCGGAGAACCTGCGTGCAATC |  |
| Internal PCR | chiKF | GGCTCTAGAGCCTCTGCTAACCATG | 0.89 kb |
|  | GFPr1 | CCTTCAGCTCGATGCGGTTCACCAG |  |
| 3’ PCR | ADB296 | AGGGGCTCGCGCCAGCCGAACTGTT | 6.5 kb |
|  | PCR4 | CTCAGTGGCTCAACAACACTTGGTC |  |
| 3’ PCR | ADB298 | GCGGCCGGAGAACCTGCGTGCAATC | 5.1 kb |
|  | PCR4 | CTCAGTGGCTCAACAACACTTGGTC |  |


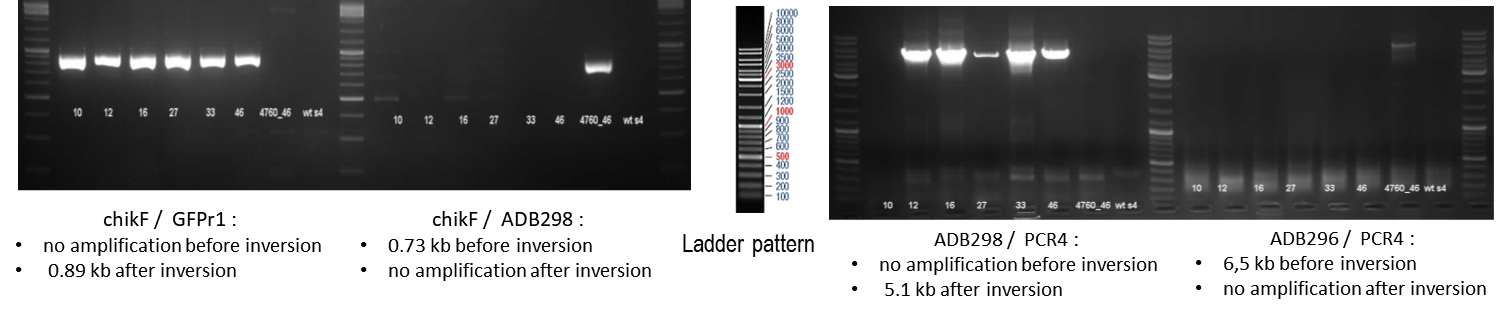
Six candidate clones identified by internal PCR screening were further analyzed by 3’ PCR screening. These 6 clones (clones #10, #12, #16, #27, #33, and #46) were confirmed for Cre inversion.


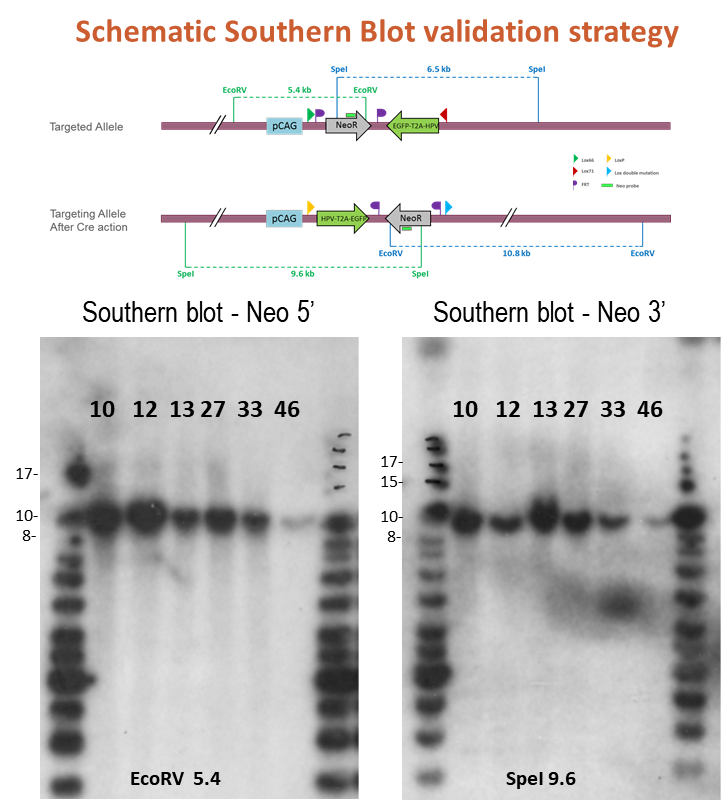


**Digestions used to validate the 5’ and 3’ insertion**

|  |  |  | Targeted Allele |  |
| --- | --- | --- | --- | --- |
| **Probe** | **Name** | **Genomic DNA digest** | **Before**  **reversal (kb)** | **After**  **reversal (kb)** |
| Neo | 5’ digest | EcoRV | 5.4 | 10.8 |
|  | 3’ digest | SpeI | 6.5 | 9.6 |

## Fluorescence imaging to evaluate expression of E7/HPV16 - T2A – eGFP cassette


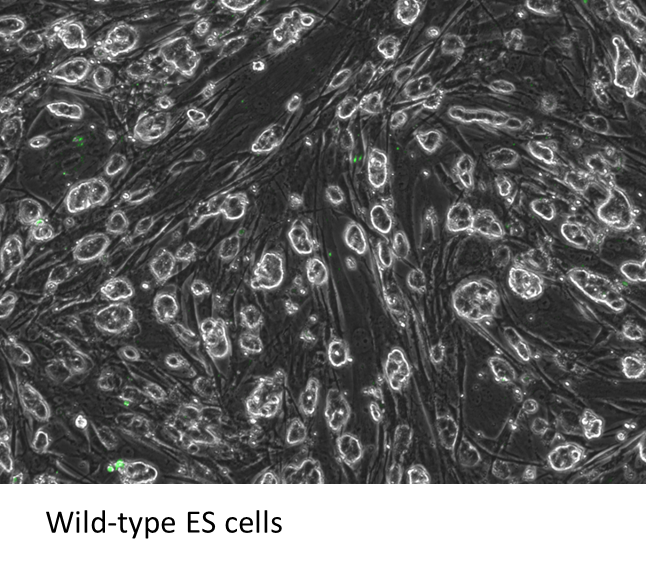


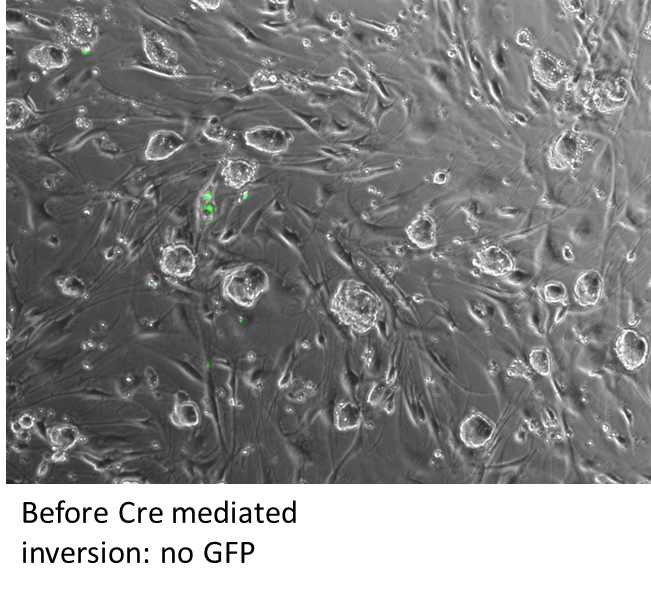


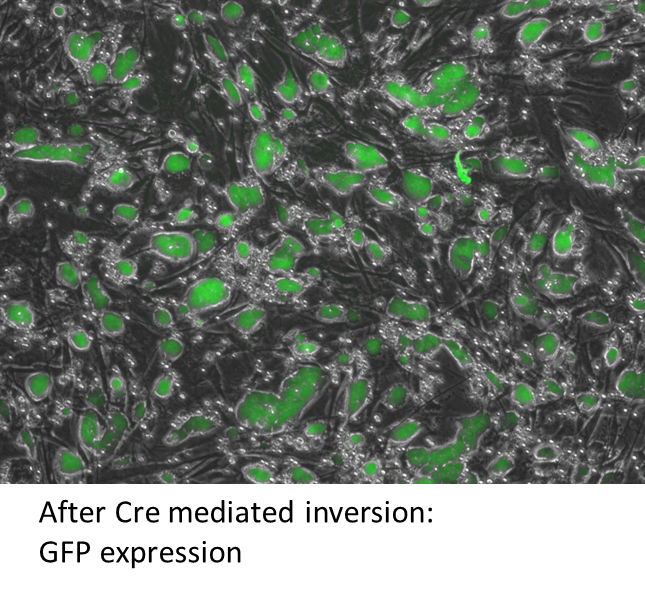


# Microinjection & chimera breeding & germline transmission

Recombinant ES clone #46 validated in the previous project phase was injected into blastocysts to generate chimeric males. Recipient blastocysts were isolated from mated BALB/cN females (Health status SPF Specific Pathogen Free). The S4 ES cells used in the injection experiment were originally derived from a C57BL/6NCrl mouse strain (which has a black coat color). These cells were injected into blastocysts derived from a BALB/cN strain, which has a white coat color. The resulting offspring are thus chimeras of two different cell types (ES cell-derived cells and host blastocyst-derived cells) and the degree of chimerism was monitored by the percentage of light and dark patches on these animals.

Four resulting chimeric males (55 to 85 % coat color) were mated with Flp deleter C57BL/6NCrl females which show maternal contribution (Birling *et al*., 2012; health status SPF – Specific Pathogen Free) to investigate whether the recombined ES cells have contributed to the germ layer. Germline transmission was obtained on week 33/2014.

All generated Gt(ROSA)26Sor^tm1(CAG‐E7,‐EGFP)Ics^ mice were always maintained on C57BL/6N pure background.

**References:**

Unidirectional Cre-mediated genetic inversion in mice using the mutant loxP pair lox66/lox71. Oberdoerffer, P. Nucleic Acids Research. 2003 31(22), pp. 140e–1140. Available at: <https://doi.org/10.1093/nar/gng140>.

Highly-efficient, fluorescent, locus directed cre and FlpO deleter mice on a pure C57BL/6N genetic background. Birling MC, Dierich A, Jacquot S, Hérault Y, Pavlovic G. Genesis. 2012 Jun;50(6):482-9. doi: 10.1002/dvg.20826. Epub 2012 Mar 20.
