# Supplementary material for: Development of HPV16 mouse and dog models for more accurate prediction of human vaccine efficacy
Source: Lab Anim Res. 2023 Jun 12;39:14. doi: 10.1186/s42826-023-00166-3 (PMC10258489; doi:10.1186/s42826-023-00166-3)
Supplement: Supplementary file 3 — Additional file 3. Genotyping protocol for E7inv line. [file 42826_2023_166_MOESM3_ESM.docx]

**Supplementary File 2- genotyping protocol for the Gt(ROSA)26Sor^tm1(CAG‐E7,‐EGFP)Ics^ line**

# Schematic map showing the position of the primers

The position of the primers is not to scale.

# Sequence of primers used for genotyping

| **Position** | **Primer code** | **Sequence** |
| --- | --- | --- |
| Ef | 5641 | GGCTCTAGAGCCTCTGCTAACCATGT |
| Er | 5736 | GTGGTTTGTCCAAACTCATC |
| Lf | 1857 | GCGGAGCCGAAATCTGGGAG |
| Lxr | 7723 | TGCTATACGAACGGTAGGCCGG |
| Sf | 7679 | TCCTCGCTGCTGTCGTT |
| Wr | 5646 | CACATGGTCCTGCTGGAGTTCGT |
| Xf | 4036 | AAAGTCGCTCTGAGTTGTTAT |
| Xr | 4035 | CCTTTAAGCCTGCCCAGAAG |

# Expected PCR fragment size for each allele (in bp)

| **Region analyzed** | **Pair of primers** | **Wild-type allele** | **E7^inv^ allele** | **E7^+^ allele** |
| --- | --- | --- | --- | --- |
| Wild-type allele-specific PCR | Xf / Xr | 239 | 3163* | 3163* |
| 5’ end of the E7^inv^ allele | Ef / Er | --- | 249 | --- |
| 3’ end of the E7^inv^ allele | Sf / Xr | --- | 389 | --- |
| 5’ end of the E7^+^ allele (**with DMSO**)^1^ | Lf / Lxr | --- | --- | 340 |
| 3’ end of the E7^+^ allele | Wr / Xr | --- | --- | 353 |

*: this amplicon is not observed using the genotyping conditions described here

---: no amplicon should be obtained

^1^ DMSO should be added to this PCR as described in composition of the PCR mix

# Composition of the PCR mix

Reagents: Volume:

- FastStart PCR Master (Roche) 7.5µl

- DNA (50ng µl^−1^) 1.5µl

- 5’ primer (100 µM) 0.06µl

- 3’ primer (100 µM) 0.06µl

- Sterile H_2_O up to 15 µl

5% of DMSO should be added to the reaction mix for Lf / Lxr PCR

# PCR cycling conditions

| **Temperature** | **Time** | **#Cycles** |
| --- | --- | --- |
| 95°C | 4min | 1 |
|  |  |  |
| 94°C | 30s |  |
| 62°C | 30s | 35 |
| 72°C | 1min |  |
|  |  |  |
| 72°C | 7min | 1 |
|  |  |  |
| 14°C | --- | --- |

Additional details on genotyping protocols and troubleshooting are described in Jacquot *et al.* 2019

Jacquot S, Chartoire N, Piguet F, Hérault Y, Pavlovic G. Optimizing PCR for Mouse Genotyping: Recommendations for Reliable, Rapid, Cost Effective, Robust and Adaptable to High‐Throughput Genotyping Protocol for Any Type of Mutation. Current Protocols in Mouse Biology. 2019;9. doi:10.1002/cpmo.65.
