# Supplementary material for: Development of HPV16 mouse and dog models for more accurate prediction of human vaccine efficacy
Source: Lab Anim Res. 2023 Jun 12;39:14. doi: 10.1186/s42826-023-00166-3 (PMC10258489; doi:10.1186/s42826-023-00166-3)
Supplement: Supplementary file 4 — Additional file 4. Table S1: Integrative and non-integrative lentivectors and administration conditions used in this study. [file 42826_2023_166_MOESM4_ESM.docx]

## Supplementary Table 1 Integrative and non-integrative lentivectors and administration conditions used in this study

| **Vectors** | **Type of delivery system** | **Titer (TU-PP ml^-1^)** | **Injected volume per animal (ml)** | **Dose (TU-PP) / point** | **Animals** |
| --- | --- | --- | --- | --- | --- |
| LentiFlash® Cre particles | Non-integrative vector | 9.1 x 10^11^ PP ml^-1^ | 0.02 | 7.4 x 10^11^ PP | 15 E7^inv^/wt mice |
|  |  | 5.1 x 10^11^ PP ml^-1^ |  |  |  |
|  |  | 6.2 x 10^11^ PP ml^-1^ | 0.02 | 1.2 x 10^10^ PP | 6 E7^inv^/wt mice (+3 NI) |
|  |  |  |  |  |  |
| E7/HPV16‐ZsGreen ILV | Integrative lentivector | 3.2 x 10^9^ TU ml^-1^ | 0.02 | 6.4 x 10^7^ TU | 3 wild-type mice |
|  |  | 3.2 x 10^9^ TU ml^-1^ | 0.05 | 1.6 x 10^8^ TU | 1 dog |
|  |  | 1.9 x 10^9^ TU ml^-1^ | 0.05 | 9.5 x 10^7^ TU | 8 dogs |
